# Early HHV-6 IgG and IgA responses during acute SARS-CoV-2 infection in relation to long COVID: a multicohort observational study

**DOI:** 10.1016/j.ebiom.2026.106455

**Published:** 2026-08-27

**Authors:** Ananya Choudhury, Michael J. Burry, Woo Joo Kwon, Xihui Yin, Alysa Rallistan, Sonia Presti, Gabrielle S. Ndakwah, Emily Q. Cheng, Muge Kalaycioglu, Marlayna Harris, Jennifer Frankovich, Elizabeth W. Karlson, Jenna Bollyky, Paul J. Utz, Tyler R. Prestwood

**Affiliations:** a Division of Immunology and Rheumatology, Department of Medicine, Stanford University School of Medicine, Palo Alto, CA, USA; b Division of Allergy, Immunology, & Rheumatology, Department of Pediatrics, Stanford University School of Medicine, Palo Alto, CA, USA; c Stanford Immune Behavioral Health Clinic and Research Program, Stanford University & Stanford Children's Health, Palo Alto, CA, USA; d Institute for Immunity, Transplantation and Infection, Stanford University School of Medicine, Palo Alto, CA, USA; e Division of General Psychiatry and Psychology, Department of Psychiatry and Behavioral Sciences, Stanford University School of Medicine, Palo Alto, CA, USA; f Division of Primary Care & Population Health, Stanford University School of Medicine, Palo Alto, CA, USA; g Department of Medicine, Division of Rheumatology, Inflammation, and Immunity, Brigham and Women's Hospital, Boston, MA, USA; h Massachusetts General Brigham, Boston, MA, USA

**Keywords:** Long COVID, SARS-CoV-2, HHV-6, Herpesvirus immunity, IgA, IgG, Antibody profiling, Biomarkers, Immune response, Translational immunology

## Abstract

**Background:**

Long COVID is a heterogeneous condition associated with both early immune responses to SARS-CoV-2 and antibody responses to herpesviruses. However, herpesvirus-directed antibody responses during acute SARS-CoV-2 infection and their relationship to subsequent long COVID remain poorly understood.

**Methods:**

We developed a multiplex bead-based serologic assay using recurrent, public peptide epitopes spanning all eight human herpesviruses. Antibody responses were profiled in longitudinal samples from acute SARS-CoV-2 infection and in early post-infection samples from participants in the NIH RECOVER observational cohort. IgG, IgA, and IgM responses were analysed using peptide-, virus-, and factor-level approaches.

**Findings:**

During acute SARS-CoV-2 infection, a subset of individuals exhibited increased herpesvirus-directed IgA responses, particularly against beta-herpesviruses, without corresponding increases in IgG or IgM. In RECOVER participants, subsequent long COVID was associated with herpesvirus- and isotype-specific enrichment of high responders, most prominently HHV-6 IgA and HHV-1/HHV-2 IgG. Unsupervised factorisation identified distinct HHV-6 IgG- and IgA-dominant antibody programs with differing clinical and demographic associations. HHV-6 IgG responses were associated with lower symptom burden and relative enrichment among participants without long COVID, whereas HHV-6 IgA responses were associated with greater symptom burden. HHV-6 IgG responses also declined with increasing age, with the association more readily detected among females.

**Interpretation:**

Early herpesvirus-directed antibody responses following SARS-CoV-2 infection exhibit distinct virus- and isotype-specific patterns associated with long COVID. These findings suggest that heterogeneity in long COVID may be linked to differential herpesvirus-directed humoural immune responses that emerge early after infection.

**Funding:**

This study was funded by Stanford Post-Acute Recovery Cohort; NIH and RECOVER grants; the Henry Gustav Floren Family Trust; the Stanford Department of Medicine Team Science Program; Stanford Institutes of Medicine Summer Research Program (SIMR); the Doris Duke Charitable Foundation; the SPARK Program; Nucleate Dojo; the Jessica Lynn Saal Memorial Award; the William and Marissa Rastetter Research Scholar Fund through the Robinson Life Sciences, Business, and Entrepreneurship Program; National Institute of Diabetes and Digestive and Kidney Diseases of the National Institutes of Health Award.

**Research in context**

**Evidence before this study**

We searched PubMed and medRxiv for studies published through June 2026 using combinations of the terms “long COVID”, “post-acute sequelae of SARS-CoV-2 infection”, “PASC”, “herpesvirus”, “human herpesvirus 6”, “HHV-6”, “Epstein–Barr virus”, “EBV”, “cytomegalovirus”, “CMV”, “reactivation”, “antibody”, “serology”, “IgG”, and “IgA”. We reviewed original research articles, cohort studies, and reviews investigating herpesvirus infection, reactivation, or humoural immune responses in COVID-19 and long COVID.

Multiple studies have reported associations between herpesvirus biomarkers and long COVID. Early investigations identified Epstein–Barr virus (EBV) reactivation during acute SARS-CoV-2 infection as a potential predictor of subsequent long COVID symptoms, while subsequent studies reported associations between serologic evidence of herpesvirus activity and persistent symptom burden. Reviews have highlighted herpesvirus reactivation as a plausible contributor to long COVID pathogenesis, although findings have varied across cohorts and direct evidence of active viral replication has been inconsistent. Most prior studies focused on EBV, viral nucleic acid detection, or whole-virus serologic measurements. Comparatively little attention has been given to human herpesvirus 6 (HHV-6), despite longstanding interest in HHV-6 in post-infectious and fatigue-associated illnesses. Furthermore, few studies have examined herpesvirus-directed antibody responses during the early period following SARS-CoV-2 infection, and none, to our knowledge, have systematically profiled epitope-resolved IgG and IgA responses spanning all eight human herpesviruses in relation to subsequent long COVID development.

**Added value of this study**

We developed an epitope-resolved multiplex serologic platform targeting recurrent antibody epitopes across all eight human herpesviruses and applied it to longitudinal cohorts spanning acute SARS-CoV-2 infection and early post-infection follow-up. This approach enabled characterisation of virus-specific and isotype-specific antibody responses at peptide resolution. During acute SARS-CoV-2 infection, a subset of individuals exhibited increased herpesvirus-directed IgA responses, particularly against beta-herpesviruses. In an independent NIH RECOVER cohort, subsequent long COVID was associated with herpesvirus- and isotype-specific enrichment of high responders, most prominently HHV-6 IgA and HHV-1/HHV-2 IgG. Unsupervised factorisation further identified distinct HHV-6 IgG- and IgA-dominant antibody programs associated with different clinical and demographic features. These findings extend prior observations linking herpesviruses to long COVID and identify previously unrecognised heterogeneity in herpesvirus-directed humoural immunity.

**Implications of all the available evidence**

Current evidence suggests that long COVID is biologically heterogeneous and may arise through multiple, partially overlapping mechanisms. Our findings support a model in which herpesvirus-directed immune responses contribute to this heterogeneity and demonstrate that these responses differ substantially by virus and antibody isotype. The identification of distinct HHV-6 IgG- and IgA-associated response patterns suggests that herpesvirus-related immune signatures may help define biologically meaningful long COVID subgroups. More broadly, these results highlight the value of epitope-resolved serologic approaches for investigating post-viral syndromes and motivate future studies examining the mechanistic relationship between herpesvirus immunity, mucosal immune responses, and long COVID pathogenesis.

## Introduction

Long COVID (LC) is a biologically mediated, post-infectious condition that can substantially disrupt health, functional capacity, and quality of life in previously healthy and high-functioning individuals.^1^, ^2^, ^3^ LC often impairs return to work, caregiving responsibilities, and activities of daily living,^2^^,^^4^^,^^5^ and has been estimated to have an economic impact of over $1 trillion.^1^ Even among individuals who experience partial recovery, the risk of reinfection or symptom recurrence can continue to limit social and occupational participation and contribute to persistent isolation.^5^, ^6^, ^7^ Because SARS-CoV-2 continues to circulate globally, LC remains a substantial public health concern even for individuals without prior comorbidities,^8^, ^9^, ^10^ underscoring the need for biologically grounded markers that can clarify disease mechanisms and identify early immune features associated with long-term outcomes.

Despite its marked clinical heterogeneity, convergent evidence supports a model in which early immune events during acute SARS-CoV-2 infection influence longer-term outcomes.^11^, ^12^, ^13^ Proposed mechanisms include persistent immune activation, altered innate and adaptive immune cell states, autoantibody production, endothelial and microvascular injury, fibrin-rich microclots, complement activation, and neuroendocrine or hormonal perturbations.^2^^,^^3^^,^^11^^,^^14^, ^15^, ^16^, ^17^, ^18^ Importantly, longitudinal studies initiating sampling during acute infection demonstrate early divergence in immune trajectories between individuals who do and do not develop LC, suggesting that risk is pre-existing or shaped near the time of primary infection rather than arising exclusively in the post-acute phase.^12^^,^^13^^,^^16^^,^^19^

Herpesviruses, particularly Epstein–Barr virus (EBV/HHV-4), have been hypothesised to contribute to these early immune trajectories.^13^^,^^16^^,^^20^ Several studies have reported associations between LC and serologic or other markers of recent EBV activity, typically measured months after acute infection.^13^^,^^16^^,^^20^^,^^21^ However, these findings have been heterogeneous and have relied largely on conventional IgG-based serology or limited viral DNA detection, which provide indirect and time-averaged views of immune activity and may miss transient or compartmentalised immune events.^15^^,^^22^ In contrast, humoural immune responses during acute SARS-CoV-2 infection are highly dynamic and isotype-specific, with IgM and IgA responses arising early and differing from IgG responses in timing, magnitude, and immunologic implications.^23^, ^24^, ^25^, ^26^, ^27^ IgA, in particular, plays a central role in mucosal antiviral immunity and may capture barrier-associated immune activation not reflected by IgG-focused profiling,^26^^,^^28^^,^^29^ raising the possibility that isotype-resolved antibody measurements during acute infection could reveal features relevant to LC risk that have not been captured by prior studies.

We hypothesised that epitope- and isotype-resolved profiling of herpesvirus-directed antibody responses during acute SARS-CoV-2 infection could identify structured immune signatures associated with subsequent LC. Antiviral humoural responses are inherently heterogeneous and are most often measured using whole-virus antigens or cell lysates prepared from infected cells, which aggregate responses across many epitopes and obscure epitope-specific patterns.^30^, ^31^, ^32^ To overcome this limitation, we developed a multiplexed, microbead-based peptide platform using a library of conserved, minimal public epitopes derived from large-scale virome-wide serologic datasets,^32^, ^33^, ^34^ with modified peptide linkers to enable robust, sensitive, and reproducible epitope-resolved antibody measurements. Applying this approach to longitudinal acute-infection cohorts and early post-infection samples from the NIH RECOVER initiative, we identify virus- and isotype-specific herpesvirus antibody response programs that emerge early after SARS-CoV-2 infection and associate with differential risk of developing LC.

## Methods

### Study design

This study used a multiplex peptide-microbead serologic platform to profile epitope-resolved IgG and IgA responses to human herpesviruses in two complementary settings: longitudinal acute SARS-CoV-2 infection cohorts and an early post-infection subset of the NIH RECOVER cohort. The primary objective was to determine whether herpesvirus-directed antibody patterns measured during or soon after acute SARS-CoV-2 infection were associated with subsequent LC. Secondary objectives were to define longitudinal antibody dynamics during acute infection and to identify coordinated antibody-response modules associated with LC risk, symptom burden, and demographic features.

### Study cohorts

#### RECOVER cohort

RECOVER participant samples were collected within 30 days of SARS-CoV-2 infection. LC status was defined using the Long COVID Research Index (LCRI) published by Geng et al.^35^ Although the original sample assignments for participants included in this study were provided using post-acute sequelae of COVID-19 (PASC)-based group labels, all analyses in the present study used a dichotomised LCRI-based classification. Participants were included for analyses as LC+ (LCRI ≥11) or LC− (LCRI 0–10). The final analytic cohort included 124 LC+ participants and 83 LC− participants. The analytic set was determined by the number of RECOVER samples available within the prespecified early post-infection window and meeting LC classification criteria, together with restriction to participants with sufficient clinical data for LCRI-based classification and practical constraints related to batch-based Luminex plate capacity; no imputation was performed.

#### CHART and TrackCOVID cohorts

The COVID-19 Healthcare Worker Antibody and Reverse Transcription PCR Tracking (CHART) and TrackCOVID cohorts were longitudinal SARS-CoV-2 surveillance studies conducted in the San Francisco Bay Area during 2020. The CHART cohort enrolled employees at three medical centres (Stanford Medical Center, UCSF Health, and Zuckerberg San Francisco General Hospital) between July and November 2020 and followed participants with biweekly SARS-CoV-2 RT-PCR testing and serial serologic sampling.^36^ The TrackCOVID cohort enrolled community-residing adults from six Bay Area counties between July and December 2020 using an address-based stratified random sampling design and followed participants with approximately monthly SARS-CoV-2 RT-PCR and serologic testing.^37^ For the present study, longitudinal biospecimens were selected from SARS-CoV-2-infected individuals and matched SARS-CoV-2-negative controls based on demographic and sampling characteristics. A total of 12 subjects from the combined CHART and TrackCOVID cohorts were analysed across five longitudinal time points spanning pre-infection, peri-infection, and post-infection sampling. In addition, two subjects were assayed but are not shown because only four of the five target time points were available. Serum or plasma samples from these cohorts were used to characterise antibody- and epitope-level immune responses associated with SARS-CoV-2 infection across healthcare-associated and community-based populations.

Detailed descriptions of the CHART, TrackCOVID, and RECOVER cohorts, including participant characteristics, sampling windows, and analytic inclusion criteria, are provided in Supplementary Table S1.

### Ethics

Samples from the RECOVER (Researching COVID to Enhance Recovery) cohort were obtained from adult participants (≥18 years) enrolled in the multicentre NIH RECOVER observational study of SARS-CoV-2 infection and its post-acute sequelae (Study ID S21-01226). All participants provided written informed consent.^38^^,^^39^ The CHART study was approved by the University of California, San Francisco Committee on Human Research and the Stanford University School of Medicine Panel on Human Subjects in Medical Research (IRB-56188). All participants provided written electronic informed consent.^36^ The TrackCOVID study was initially designated as a public health surveillance activity under 45 CFR 46.102(l) by the Stanford University School of Medicine Administrative Panel on Human Subjects in Medical Research and the University of California, San Francisco Institutional Review Board, with written informed consent obtained from all participants.^37^ Samples subsequently collected for research purposes during the TrackCOVID continuation phase were obtained under Stanford IRB 62981 (“A Continuation Study of the Incidence and Seroprevalence of the Novel Coronavirus in the Bay Area: TrackCOVID Breakthrough Study”). All studies were conducted in accordance with the principles of the Declaration of Helsinki and applicable institutional and federal guidelines.

### Reagent validation

All antibodies, peptide reagents, commercial antigens, and assay reagents used in this study were obtained from commercial sources or synthesised by approved vendors, with the exception of mouse anti-FLAG M1 monoclonal antibody which was generously gifted by the Kobilka lab. Detailed reagent information, including vendor names, catalogue numbers, and Research Resource Identifiers (RRIDs) where available, is provided in Reagent Validation File. The complete herpesvirus peptide panel, including peptide identifiers, protein annotations, and epitope sequences, is provided in Supplementary Table S2. Commercial viral antigens used for assay development and validation are listed in Supplementary Table S3. Secondary-antibody performance was evaluated using dilution-series experiments designed to assess isotype specificity, minimise cross-reactivity, and optimise assay signal-to-noise characteristics. Representative validation data for IgA and IgM detection are shown in Supplementary Fig. S1.

### Peptide selection and assay design

Peptides were selected based on previously identified immunodominant and recurrent (“public”) herpesvirus epitopes derived from high-throughput phage-display serological profiling (VirScan) datasets.^32^^,^^34^ Candidate peptides were prioritised based on consistent seroreactivity across independent cohorts, enrichment in individuals with prior herpesvirus exposure, and representation across multiple human herpesviruses. The final peptide panel comprised epitopes derived from eight human herpesviruses, including Epstein–Barr virus (EBV), cytomegalovirus (CMV), and additional herpesvirus family members. Scrambled (SCR) peptides, which were derived from authentic peptide epitopes in the array, were included to estimate nonspecific binding and enable sample-level background correction. All peptides were synthesised at ≥40% purity (GenScript) with a modified C-terminus containing a β-alanine linker, a poly-lysine spacer, and a terminal cysteine to facilitate covalent coupling to carboxylated beads.^40^ Peptides also incorporated an N-terminal FLAG tag to enable validation of peptide immobilisation. Peptide sequences, viral sources, and associated annotations are provided in Supplementary Table S2.

Analytical RP-HPLC peak tables were available for all 86 peptides. The largest integrated 220-nm UV peak area percentage was recorded as a conservative HPLC purity metric, yielding a mean of 51.4% and median of 50.1% across peptides. Because several chromatograms showed apparent peak splitting, shoulders, or broad unresolved elution, we also calculated a descriptive apparent product-region area by summing contiguous peak bins within the dominant elution region; this adjusted product-region estimate had a mean of 74.1% and median of 78.4%. These adjusted product-region values were used only as a descriptive QC metric and were not interpreted as definitive chemical purity, because adjacent or broadened HPLC peaks may include closely related synthesis byproducts in addition to product-associated species.

### Peptide conjugation to beads

Antibody profiling was performed using a magnetic microbead-based multiplex assay adapted from established bead-based serologic immunoassay protocols, with modifications to accommodate peptide antigens and reduce nonspecific binding.^40^, ^41^, ^42^ During method development, three peptide immobilisation strategies were evaluated: standard EDC/Sulfo-NHS carbodiimide coupling, adipic acid dihydrazide (ADH)-mediated spacer-based conjugation, and antibody-mediated immobilisation via N-terminal FLAG tags. Across validation experiments, ADH-mediated conjugation produced higher and more reproducible mean fluorescence intensity (MFI) values than direct carbodiimide coupling or antibody-mediated immobilisation and was therefore selected for all subsequent experiments.

Peptides were synthesised with terminal modifications to improve solubility, support bead coupling, and enable display validation. FLAG-tagged peptides were assessed using a mouse anti-FLAG M1 monoclonal antibody (generously gifted by the Kobilka lab). Antigen specificity was further evaluated using competitive inhibition assays in which sera were preincubated with excess soluble cognate peptide before bead incubation. Peptide-only and bare-bead controls were included to define baseline fluorescence. For the final assay, peptides were covalently coupled to carboxylated magnetic microspheres (Luminex Corporation, Austin, TX, USA; MagPlex microspheres, Cat# MC100XXX-01) using adipic acid dihydrazide (ADH; Santa Cruz Biotechnology, Dallas, TX, USA; Cat# sc-257072)-mediated conjugation adapted from standard carbodiimide chemistry employing 1-ethyl-3-(3-dimethylaminopropyl)carbodiimide hydrochloride (EDC; Thermo Fisher Scientific, Waltham, MA, USA; Cat# 22980) and N-hydroxysulfosuccinimide sodium salt (Sulfo-NHS; Thermo Fisher Scientific, Waltham, MA, USA; Cat# 24510), followed by pooling and batch storage in StabilGuard Immunoassay Stabiliser (Surmodics, Eden Prairie, MN, USA; Cat# SG01) at −80 °C protected from light before use in multiplex profiling.^40^^,^^42^

### Antibody profiling

Following validation of bead conjugation, an 87-plex panel comprising herpesvirus-derived peptides and scrambled control peptides was used for antibody profiling. Beads were pooled and diluted to yield approximately 50 beads per peptide per well, and 5 μL of the bead mixture was aliquoted into 384-well plates. Serum samples from study participants, along with serum samples previously confirmed to be seronegative for CMV or EBV were included as biological negative controls.^43^ Samples were diluted 1:100 in StabilGuard, and 45 μL of diluted sample was added to each well. Plates were incubated at room temperature with gentle shaking, followed by three washes with PBS containing 0.05% Tween-20 using an automated plate washer. Beads were incubated with PE-conjugated goat anti-human IgG (Jackson ImmunoResearch, West Grove, PA, USA; Cat# 109-116-098; RRID:AB_2337678; 1:1000), PE-conjugated goat anti-human IgM (SouthernBiotech, Birmingham, AL, USA; Cat# 2020-09; RRID:AB_2795606; 1:200), or PE-conjugated goat anti-human IgA (SouthernBiotech, Birmingham, AL, USA; Cat# 2050-09; RRID:AB_2795707; 1:200). After washing, beads were resuspended and acquired on a Luminex FlexMap3D instrument to quantify antibody reactivity as MFI for each bead ID and corresponding peptide.

### Luminex array data processing

#### Background thresholding per peptide

To establish peptide-specific reactivity thresholds, we trained an isolation-forest background model using CMV (HHV-5) peptide responses from subjects determined to be CMV seronegative by clinical assay,^43^ together with a broader cohort containing mixed CMV serostatus. Features derived from each peptide's negative distribution (including the isolation-forest offset and the inferred log-scale background boundary) were input into a pretrained random forest regressor to generate raw MFI thresholds for each peptide in the panel. Overall, thresholds clustered between 1000 and 3000 MFI. Given this was consistent with our laboratory's historical experience in analysing Luminex array data, we set 3000 MFI as a conservative minimum floor for defining peptide reactivity.

#### Background thresholding per sample

For each sample, nonspecific binding was corrected by subtracting the minimum signal observed across three scrambled control peptides, with resulting negative values clipped to zero. Scrambled controls were derived from the same epitope library but computationally shuffled to reduce sequence identity to <40% relative to their parent peptides while preserving amino acid composition and physicochemical properties. Epitope-level reactivity was defined as signal exceeding a peptide-specific threshold after background subtraction. This conservative, subject-specific normalisation minimises spurious or cross-reactive signal and ensures that retained responses reflect antibody reactivity above each individual's nonspecific baseline.

#### Dispersion filtering of peptides

To exclude peptides with rare or unstable signals, we applied a dispersion filter to the primary analytical cohorts. For each peptide, we quantified (i) cohort representation as the number of reactive subjects (*n_pos*); (ii) contributor dispersion using an effective number of independent contributors, (*N*_*eff*_ = (∑*w*)^2^/∑*w*^2^), where *w* denotes the positive excess MFI above the background threshold; and (iii) outlier dominance as the fraction of total signal attributable to the strongest subject, (*top share* = *max(w)/*∑*w*). The *N*_*eff*_ term is a concentration-derived effective-number quantity related to formulations used in diversity analysis to distinguish broadly distributed from highly concentrated contributions.^44^^,^^45^ Peptides were excluded if they showed low representation (*n_pos* < 4), low dispersion (*N*_*eff*_ < 20), or outlier-dominated signal (*top share* > 0.15). Filtering was performed on the IgG dataset to define a stable, cohort-representative peptide panel, which was then applied unchanged to the IgA dataset for visualisation and analysis. This approach ensured that IgA responses were evaluated within a peptide space validated for background and dispersion properties in the higher-sensitivity IgG assay. Scrambled peptides were excluded from filtering.

### Integrated upper-tail quantile differences

To quantify group differences concentrated among high-responding individuals rather than shifts in central tendency, we applied a quantile-based distributional analysis to dispersion-filtered, background-subtracted, and thresholded peptide reactivity values. This approach was motivated by recent biological applications of quantile analysis that identify heterogeneous effects across the phenotype distribution, including effects concentrated in upper-risk subgroups, but was adapted here to summarise upper-tail differences in peptide reactivity between groups.^46^ For each peptide, group separation was measured as the difference in peptide reactivity between groups at a given quantile of the distribution, denoted *ΔQ*(*q*). Because cohort sizes were modest and signals appeared concentrated in the upper tail, we summarised these effects using an integrated upper-tail metric. Specifically, *ΔQ*(*q*) values were averaged across progressively stricter upper-tail intervals ending at *q* = 0.97, yielding integrated quantile shifts (*iΔQ*). Intervals evaluated included 50–97%, 70–97%, 80–97%, and 90–97%. The upper bound of 97% was chosen as the highest quantile supported by the available sample sizes after filtering, corresponding to approximately 4–5 LC+ and 2–3 LC− samples contributing to the extreme tail.

### Peptide-level upper-tail enrichment analysis

To identify individual epitopes for which LC+ was associated with a greater burden of high-amplitude responders, we examined peptide-level reactivity distributions in the dispersion-filtered, background-subtracted MFI values. Peptides were first ranked within each isotype by integrated upper-tail quantile shift (*iΔQ*), calculated as described above, and the top five peptides per isotype were selected for visualisation. To test whether these differences reflected enrichment in the upper tail of the distribution rather than a shift in central tendency, we applied a one-sided tail-focused Anderson-Darling permutation test at the individual peptide level. This statistic was chosen as it emphasises group differences among the highest-responding individuals while retaining the full continuous signal distribution. Significance was assessed using 20,000 label permutations under the alternative hypothesis of greater upper-tail signal in LC+ than LC−. Benjamini-Hochberg false discovery rate (FDR) correction was applied across the displayed peptides within each isotype. This peptide-level tail analysis was used as a complementary inferential summary of the upper-tail enrichment observed in the quantile-based ranking framework.

### Virus-level upper-tail antibody enrichment analysis

The abundance of high antibody responders across peptides from each herpesvirus was compared between groups using a peptide-normalised, virus-level upper-tail framework applied to dispersion-filtered, background-subtracted peptide matrices (IgG and IgA analysed separately). Within each isotype, a peptide-specific upper-tail threshold was defined as the pooled 90th percentile of the peptide distribution across LC+ and LC− subjects. To avoid inflation from very low-amplitude peptides, upper-tail events were counted only when values exceeded both the peptide-specific threshold and an absolute background-subtracted signal floor of 3000 MFI. For each subject and virus, we then calculated the fraction of the peptides for each virus exceeding these criteria, yielding a subject-level virus score that reflects the burden of upper-tail reactivity while accounting for peptide-specific dynamic range. Group differences were summarised as the difference in mean subject-level virus scores between LC+ and LC− groups. Significance was assessed by permuting LC labels at the subject level (20,000 permutations; one-sided “greater”). Benjamini-Hochberg false discovery rate (FDR) correction was applied across the eight viruses within each isotype. The heatmap displays the difference in mean subject-level percentage above threshold, together with corresponding BH-FDR *q*-values; significance levels are indicated as ∗ *q* ≤ 0.05.

### Non-negative matrix factorisation of herpesvirus epitope responses

Non-negative matrix factorisation (NMF) was applied separately to the IgG and IgA peptide-reactivity matrices to identify latent covariance structures under non-negativity constraints. This approach was motivated by prior applications of NMF to molecular pattern discovery in high-dimensional biological data, with repeated random initialisations used to assess convergence robustness and factor stability.^47^ Each matrix consisted of samples (rows) by herpesvirus peptide epitopes (columns), with values representing excess signal above subject-specific background. Only peptides retained after dispersion and outlier filtering were included to ensure stable covariance estimation; scrambled control peptides were excluded. Each matrix was decomposed as sample-level factor scores and factor-specific peptide loadings. Factorisation was performed using standard multiplicative-update NMF with non-negativity constraints, minimising Frobenius reconstruction error. Multiple random initialisations were used to ensure convergence and stability. The number of factors (K = 4–12) was evaluated based on reconstruction error, marginal gains in enrichment metrics, and factor stability across initialisations. Improvements plateaued at K ≈ 6–7 with diminishing returns beyond K = 8, while higher values (K ≥ 9) produced increasingly diffuse factors without added biological resolution; therefore, K = 8 was selected as an optimal and reproducible solution. To assess associations with LC outcomes, sample-level factor scores were dichotomised based on upper-tail enrichment (≥90th percentile). These plots show the relative risk of LC+ among individuals in the upper tail (≥90th percentile) of each factor score distribution.

### Clinical symptoms and demographic associations of antibody response factors

Clinical symptoms were defined using RECOVER clinical data fields mapped to the analysed symptom categories. LC status for the present study was based on the revised Long COVID Research Index (LCRI), with participants dichotomised as LC+ (LCRI ≥11) or LC− (LCRI 0–10).^35^ Individuals were classified as having LC using the same symptom-based criteria applied in the primary RECOVER analyses.^38^^,^^39^ To characterise clinical features of LC associated with the major aspects of the herpesvirus antibody response, we focused on the top 12 individuals with the strongest expression of each non-negative matrix factorisation (NMF)-derived factor, representing individuals in whom the corresponding antibody program was most prominent yet mostly distinct from the other factors. Analyses were restricted to factors showing the largest relative risk differences for LC in factor-level enrichment analyses.

Symptom prevalence within each factor-enriched subgroup was calculated as the proportion of individuals reporting each symptom category. These analyses were descriptive in nature to help associate general symptom patterns, rather than to test individual symptom associations. Demographic characteristics, including age, sex at birth, and race/ethnicity, were summarised for each subgroup using descriptive statistics. Sex at birth and race/ethnicity were self-reported by participants at study enrolment. Age is reported as median and interquartile range, and sex-at-birth and race/ethnicity distributions were compared qualitatively across factors due to limited subgroup sizes.

To assess the overall demographic patterns in all subjects, continuous factor scores and age were examined across the full cohort using Spearman rank correlation. Correlations were computed across all individuals with available data and stratified by sex at birth. Statistical significance was assessed using two-sided tests, and p values were adjusted for multiple testing across factors using the Benjamini-Hochberg FDR procedure to yield adjusted *q* values (*q* ≤ 0.05 considered statistically significant).

### Statistics

Statistical analyses were performed in Python using standard scientific computing libraries. Our primary hypothesis was that long COVID would be associated with enrichment of high-reactivity responders rather than a uniform shift in antibody responses across the entire population.^11^ Accordingly, peptide- and virus-level group differences were evaluated using one-sided permutation-based upper-tail enrichment analyses designed to detect overrepresentation of long COVID-associated responses among individuals with the highest antibody reactivity. A one-sided framework was selected because the prespecified biological hypothesis was directional, testing whether long COVID was associated with increased upper-tail reactivity rather than depletion of high responders.

Permutation testing was used because antibody response distributions were frequently non-normal, right-skewed, and characterised by heterogeneous variance, with signals concentrated in a subset of highly reactive individuals. Under these conditions, tests based on shifts in central tendency, such as t-tests, may be insensitive to biologically meaningful upper-tail effects. Similarly, Wilcoxon rank-sum tests are designed to detect differences across the full distribution rather than enrichment among a limited subset of high responders. Permutation-based approaches therefore provided a distribution-free framework tailored to the primary study hypothesis.

Robustness of the main findings was assessed through concordance across complementary peptide-level, virus-level, and factorisation-based analytical frameworks. Correlations between continuous factor scores and age were evaluated using Spearman rank correlation, including sex-stratified analyses where indicated. Multiple-testing correction was performed using the Benjamini–Hochberg false discovery rate procedure, with *q* ≤ 0.05 considered statistically significant. Descriptive symptom analyses were used for exploratory interpretation and were not employed for formal testing of individual symptom associations.

### Role of funders

The funders had no role in study design, data collection, data analysis, data interpretation, or writing of the report.

## Results

### Herpesvirus minimal public epitope library

**Fig. 1:**
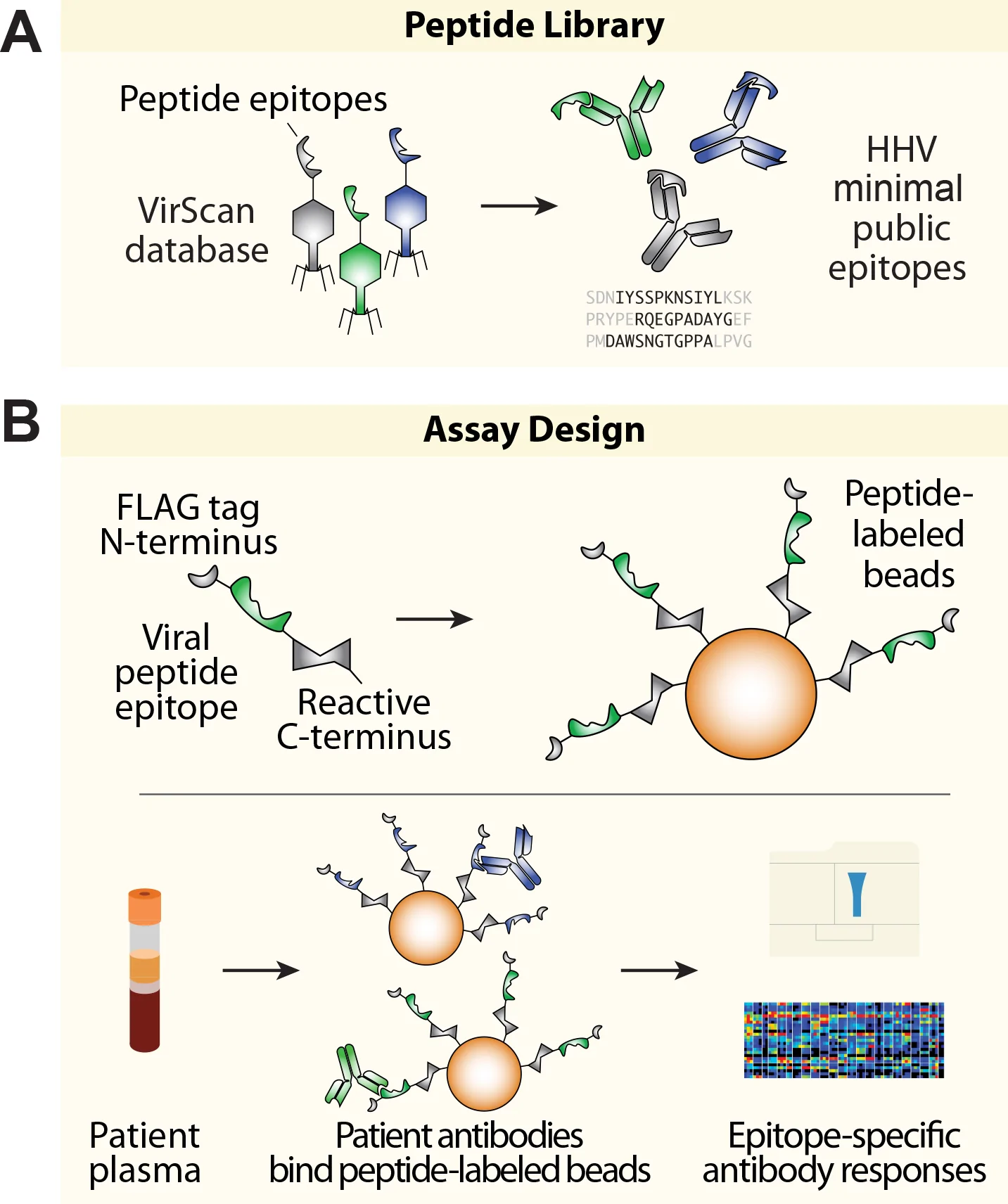
**A schematic of the minimal public-epitope herpesvirus peptide array design.** (A) Public, recurrent linear antibody epitopes were curated from large-scale VirScan datasets and collapsed to a nonredundant set spanning all eight human herpesviruses (HHV-1 to HHV-8). Overlapping and immediately adjacent epitopes were merged to reduce redundancy while preserving dominant antibody targets. (B) Peptide design and bead conjugation strategy. Each peptide was synthesised with N-terminal and C-terminal hydrophilic modifications, including an N-terminal FLAG tag for solubility and quality control and a C-terminal polylysine spacer with terminal cysteine for adipic acid dihydrazide (ADH)-mediated covalent coupling to carboxylated magnetic microbeads.

Large-scale serologic profiling studies have shown that antibody reactivity across the human virome is often concentrated within a restricted set of recurrent linear peptide epitopes shared across individuals, often referred to as public epitopes.^32^^,^^33^ Subsequent refinement of these datasets in North American cohorts showed that a limited number of conserved peptide targets account for a substantial fraction of antiviral antibody responses, enabling construction of enriched libraries that retain high informational content while minimising low-yield features.^34^ We curated a library of the most frequently targeted linear antibody epitopes across the human virome, collapsing neighbouring or overlapping sequences into a non-redundant set of 265 peptides (15–40 amino acids in length), spanning 46 viral species, and then selected peptides corresponding to all eight human herpesviruses (HHV-1 through HHV-8). Further collapsing of overlapping herpesvirus epitopes yielded a final set of 84 non-redundant herpesvirus-derived peptide targets (Fig. 1A; Supplementary Table S2). Unlike whole-virus, infected-cell lysate, or protein-fragment-based assays, which tend to aggregate distinct responses into single summative measurements, this highly curated, epitope-resolved approach focuses on antibody responses measured at the level of individual conserved epitopes and is designed to maximise specificity and statistical power by reducing low-information antigenic content.

### Hydrophilic peptide tags improve coupling and quality control

**Fig. 2:**
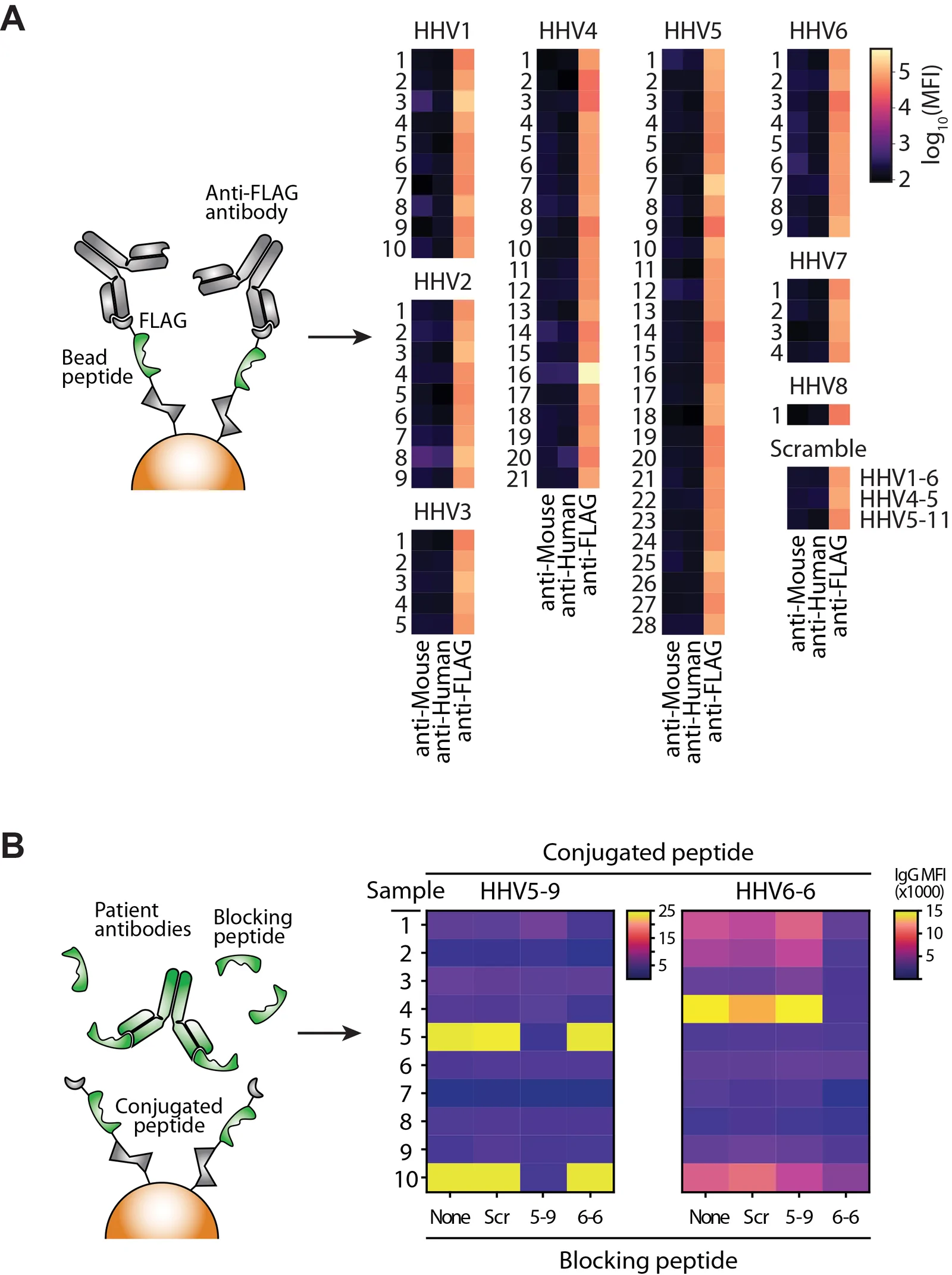
**Validation of epitope-resolved herpesvirus antibody detection.** (A) Validation of peptide immobilisation and coupling consistency. Peptides synthesised with N-terminal FLAG and C-terminal polylysine/cysteine modifications were covalently coupled to Luminex microbeads using ADH-mediated carbodiimide chemistry. Bead-bound peptides were detected using monoclonal anti-FLAG antibody followed by PE-conjugated secondary antibody, demonstrating robust and uniform signal across peptides. Minimal background was observed with secondary antibody alone. Each numbered row represents one individual peptide (total peptides per virus given in Supplementary Table S2); rows are not biological replicates. (B) Antigen specificity of peptide-antibody interactions assessed by competitive inhibition. Representative HHV-5 and HHV-6 peptides were probed with plasma samples from 10 paediatric healthy control plasma samples after preincubation with excess soluble cognate peptide, noncognate herpesvirus peptide, irrelevant peptide, or no peptide. Binding was selectively inhibited only by the cognate peptide, confirming epitope-specific antibody detection.

To profile antibody responses to these herpesvirus epitopes, we developed a peptide-based Luminex microbead array (Fig. 1B). Because coupling efficiency and nonspecific binding can be limiting for small peptide antigens,^40^, ^41^, ^42^ we modified peptide design and coupling chemistry to improve solubility, bead immobilisation, and antigen accessibility. Peptides were synthesised with terminal hydrophilic modifications, including an N-terminal FLAG epitope for display validation and a C-terminal coupling motif to support covalent attachment through an ADH-spacer strategy. All synthesised peptides dissolved readily in aqueous solution without additional solubilisation steps. Using anti-FLAG detection, all conjugated peptides showed robust signal with minimal secondary-antibody background (signal increased roughly between 100 and 1000-fold compared to secondary alone controls), confirming consistent immobilisation across the peptide set (Fig. 2A).

### Epitope-specific antibody detection with peptide-microbeads

To assess specificity of antibody detection in clinical samples, representative epitopes from HHV-5 (HHV5-9) (CMV) and HHV-6 (HHV6-6) were conjugated to Luminex beads and probed with paediatric human plasma samples. Binding specificity was evaluated using competitive inhibition assays in which plasma samples were pre-incubated with excess soluble cognate peptide, other herpesvirus peptide, irrelevant peptide, or no peptide prior to bead incubation with conjugated beads. For both selected HHV-5 and HHV-6 peptides, antibody binding to peptide-conjugated beads was selectively inhibited only by excess cognate peptide with no reduction observed following incubation with other peptides (Fig. 2B). These findings confirm antigen-specific antibody detection and are consistent with prior studies demonstrating competitive inhibition as a key control for Luminex-based serologic assays.^41^ Additional negative control samples, including CMV-seronegative donors and paediatric samples collected prior to EBV seroconversion, demonstrated minimal background signal (consistently under MFI 3000).

### IgG rapidly dominates SARS-CoV-2 antibody responses

**Fig. 3:**
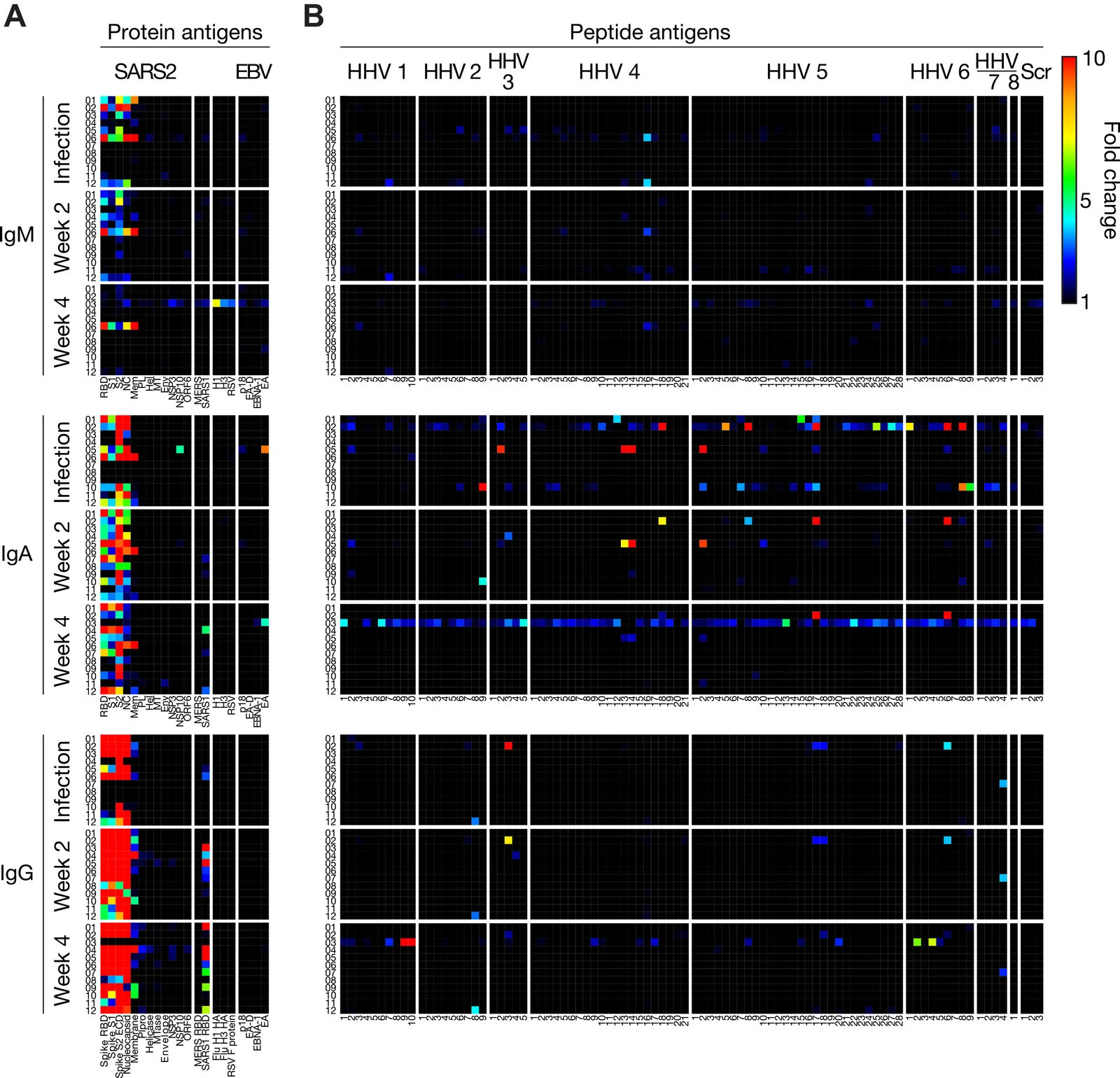
**Acute SARS-CoV-2 infection induces herpesvirus IgA responses.** Data are from 12 participants from the CHART and TrackCOVID longitudinal acute-infection cohorts with paired pre-infection and post-infection samples available for analysis. (A) Longitudinal antibody responses to SARS-CoV-2, related coronaviruses, influenza, RSV, and EBV protein antigens during acute infection and early convalescence. IgM, IgA, and IgG responses are shown at three time points: at infection, week 2, and week 4. Values are shown as fold change relative to pre-infection baseline. (B) Epitope-resolved herpesvirus antibody responses during acute SARS-CoV-2 infection. Heat maps show fold-change values relative to pre-infection baseline for IgM, IgA, and IgG across HHV-1 to HHV-8 peptides and scrambled peptide controls. IgA responses to beta-herpesvirus peptides are selectively enriched in a subset of individuals early after infection.

To validate longitudinal sampling and assay performance, we first characterised antibody responses to protein antigens from SARS-CoV-2, related coronaviruses, influenza and EBV using a traditional Luminex microbead assay (Fig. 3). The SARS-CoV-2 protein panel included canonical structural antigens—spike receptor-binding domain (RBD), spike S1, spike S2 ectodomain, nucleocapsid, and membrane—as well as additional viral proteins reported to elicit antibody responses in some individuals, including papain-like protease (PLpro), helicase (NSP13), envelope, methyltransferase (NSP14), NSP3, NSP10, and ORF6 (Fig. 3A). Antigens from SARS-CoV-1, MERS-CoV, and EBV were also included. Antibody measurements were normalised to the higher MFI of the two pre-infection time points and examined across IgM, IgA, and IgG at the first sample collected after detected infection (“Infection”), approximately two weeks later (“Week 2”), and four weeks later (“Week 4”) (Fig. 3A).

IgM responses to SARS-CoV-2 protein antigens were detected in approximately half of individuals at the first post-infection time point (Fig. 3A, IgM, Infection). These responses were heterogeneous in magnitude and antigen specificity and declined rapidly over time, with minimal IgM reactivity detectable by Week 4 (Fig. 3A, IgM, Week 4), consistent with expected early antiviral IgM kinetics.^25^^,^^26^

IgA responses were detectable early following infection and showed a more uniform pattern across individuals compared with IgM (Fig. 3A, IgA, Infection). By Week 2, all individuals exhibited detectable IgA responses to at least one SARS-CoV-2 antigen, most frequently targeting spike S2, followed by RBD and spike S1, with more limited responses to nucleocapsid (Fig. 3A, IgA, Week 2). These responses persisted through Week 4, consistent with prior observations of early IgA induction during SARS-CoV-2 infection.^24^^,^^26^^,^^48^

IgG responses were already detectable in several individuals at the first post-infection time point and increased substantially by Week 2 (Fig. 3A, IgG). By Week 2, all individuals exhibited robust IgG responses against multiple dominant SARS-CoV-2 antigens, which remained largely stable through Week 4, indicating establishment of a mature systemic humoural response.^25^^,^^49^ No consistent antibody increases were observed against EBV protein antigens during acute infection (Fig. 3A), and antibody responses to unrelated respiratory viruses (influenza and RSV) remained stable, providing evidence of detection of antigen specific antibody responses. Two subjects showed increased IgA reactivity with EBV EA antigen at single time points (Fig. 3A, IgA, Infection and Week 4), and one of those (Subject 03) also showed loss of detectable IgA and IgG against SARS-CoV-2 at Week 4, corresponding to an increase in HHV1 IgG (described in more detail below).

### SARS-CoV-1 cross-reactive IgG emerges later

IgG reactivity to SARS-CoV-1 protein antigens was not detected during the early phase of infection but became apparent only by Week 2 in some subjects and increased by Week 4 in most individuals (Fig. 3A, IgG, Week 4). In contrast, robust IgG responses to SARS-CoV-2 antigens were already established in many by the initial time point and present in all subjects by Week 2 (Fig. 3A, IgG, Week 2). IgG responses to MERS-CoV antigens remained minimal across all time points. This delayed appearance of SARS-CoV-1-reactive IgG is consistent with affinity maturation and clonal evolution of SARS-CoV-2-specific B cell responses, resulting in increased recognition of conserved epitopes shared between SARS-CoV-2 and SARS-CoV-1. This aligns with prior longitudinal studies demonstrating progressive somatic hypermutation and increasing antibody breadth following SARS-CoV-2 infection.^23^^,^^25^^,^^49^

### Acute SARS-CoV-2 infection increases beta-herpesvirus IgA

We next examined epitope-resolved antibody responses to herpesvirus peptides, rather than whole-protein antigens, during acute SARS-CoV-2 infection. Using the curated peptide library described above, we profiled IgM, IgA, and IgG responses to peptides spanning HHV-1 through HHV-8, alongside scrambled controls (Fig. 3B). Across individuals and time points, IgM and IgG responses to herpesvirus peptide epitopes were sparse and low in magnitude (Fig. 3B, IgM and IgG), consistent with the minimal serologic evidence of EBV reactivation observed at the protein antigen level (Fig. 3A), except for IgA elevation to EBV EA in two subjects described above.

In contrast to IgM and IgG, elevation of IgA responses to herpesvirus peptides was more frequently observed particularly at the initial SARS-CoV-2 time point (Fig. 3B, IgA). These responses were selectively enriched for beta-herpesviruses, particularly HHV-4, HHV-5, and HHV-6 in a subset of individuals. For each of these individuals, IgA reactivity was detectable against one or more specific epitopes from multiple herpesviruses. Responses to alpha- and gamma-herpesviruses were comparatively limited. Scrambled control peptides showed no consistent signal. Importantly, herpesvirus-directed IgA responses emerged in the context of robust contemporaneous IgA responses to SARS-CoV-2 protein antigens (Fig. 3A), without parallel increases in herpesvirus-specific IgM or IgG, with one exception (Subject 02). Furthermore, many of the epitopes showed reduced IgA reactivity by Week 4. This isotype-restricted pattern, targeting multiple herpesvirus epitopes without parallel IgG expansion, is less consistent with classical systemic herpesvirus reactivation and may reflect localised herpesvirus antigen exposure and/or bystander immune activation during acute SARS-CoV-2 infection.^26^

### Early herpesvirus antibody responses associate with long COVID

Given the early elevation of herpesvirus-directed IgA responses observed during acute SARS-CoV-2 infection, we next asked whether herpesvirus-directed antibody responses were associated with the subsequent development of LC. To address this question, we analysed samples from the NIH RECOVER biobank, focussing on individuals whose earliest available specimen was collected within 30 days of confirmed SARS-CoV-2 infection. Participants were classified as LC+ or LC− using the revised LCRI-based definition, and LC+ participants were compared with infected LC− controls.

**Fig. 4:**
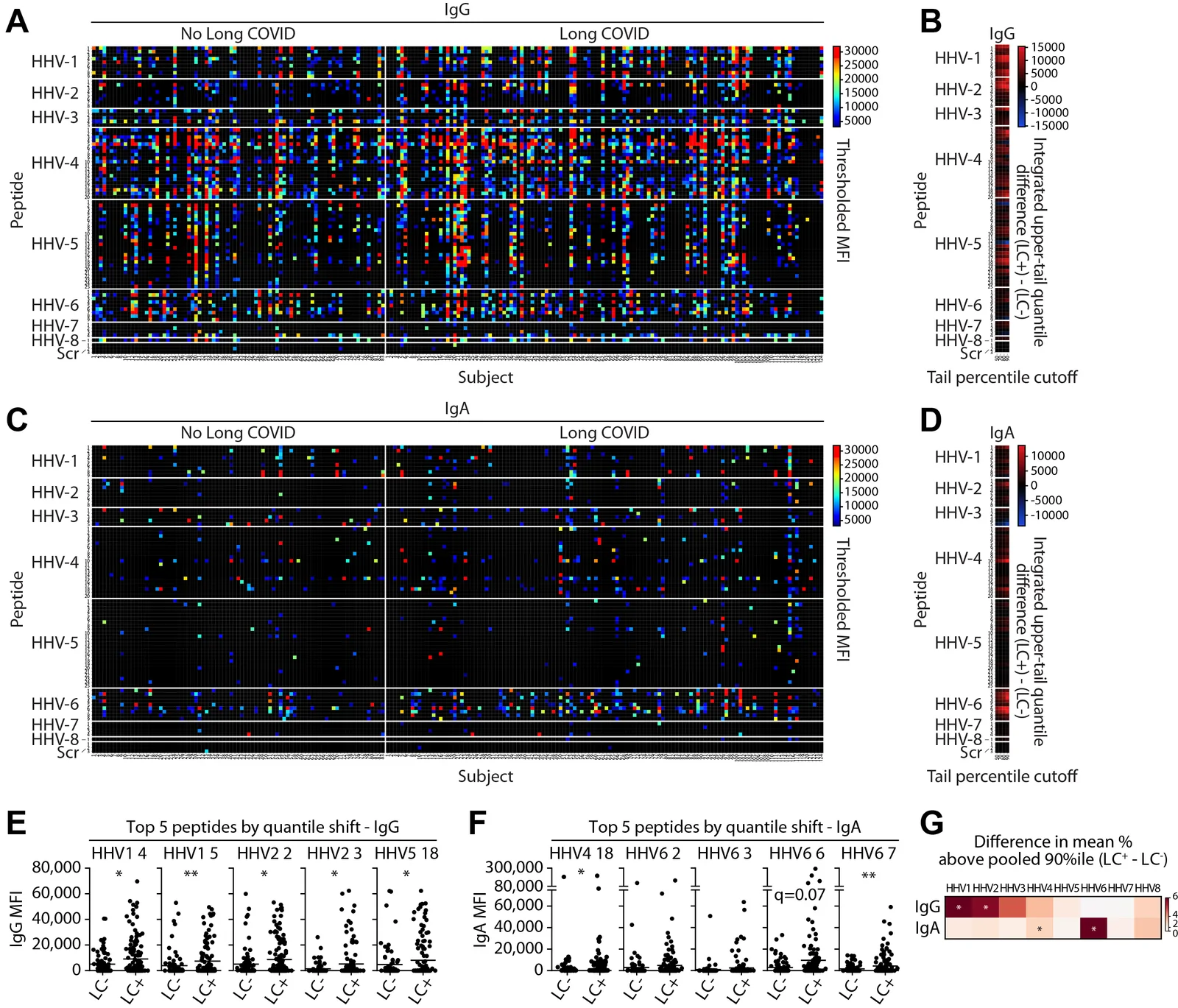
**Virus- and isotype-specific herpesvirus antibody enrichment in long COVID.** Data are from the RECOVER analytic cohort (124 LC+ and 83 LC− participants). (A) Heat map of background-subtracted IgG responses to herpesvirus peptides in individuals with long COVID (LC+) and controls without long COVID (LC−). Rows correspond to peptides grouped by viral species; columns represent individual subjects. (B) Integrated upper-tail quantile differences (LC+ − LC−) for IgG responses across peptides and tail percentile cutoffs, highlighting enrichment among high responders rather than shifts in central tendency. (C) Heat map as in (A), but for IgA responses. IgA reactivity was sparse overall but showed selective enrichment for HHV-6 peptides in LC+ subjects. (D) Integrated upper-tail quantile differences for IgA responses, demonstrating virus-specific enrichment concentrated among HHV-6 peptides. (E) Distribution of IgG MFI values for the top five peptides ranked by integrated upper-tail quantile shift. Each point represents an individual subject. Significance was assessed using a one-sided tail-focused Anderson-Darling permutation test comparing LC+ and LC− distributions, with Benjamini-Hochberg false discovery rate correction across displayed peptides (∗ *q* ≤ 0.05, ∗∗ *q* ≤ 0.01). (F) Distribution of IgA MFI values for the top five peptides ranked by integrated upper-tail quantile shift, as in (E). The exact q value is given for comparisons approaching but not meeting the significance threshold. (G) Virus-level upper-tail antibody enrichment for IgG and IgA. Heat map colour indicates the difference between LC+ and LC− in the mean subject-level percentage of peptides exceeding both the pooled peptide-specific 90th percentile threshold and an absolute background-subtracted signal floor of 3000 MFI, shown separately for each herpesvirus and isotype. Significance was assessed by subject-label permutation (20,000 permutations; one-sided greater) with Benjamini-Hochberg false discovery rate correction across viruses. Asterisks denote significance (q ≤ 0.05).

Using the herpesvirus public-epitope peptide Luminex array described above, we profiled IgG and IgA responses across all eight human herpesviruses. Because pre-infection baseline samples were not available for this cohort, analyses focused on absolute antibody signal magnitude (MFI) as a proxy for relative antibody abundance across subjects and peptide targets, following conservative background subtraction, dispersion filtering, and thresholding. Visual inspection of peptide-level antibody distributions revealed heterogeneity across subjects and herpesvirus species (Fig. 4A and C, Supplementary Table S4). For IgG, overall antibody abundance broadly tracked known seroprevalence patterns, with the highest reactivity observed for EBV (HHV-4) and HHV-6, followed by CMV (HHV-5), HHV-1, and HHV-3, and lower overall signal for HHV-2 and HHV-8 (Fig. 4A). IgG responses to HHV-7 were comparatively sparse despite near-universal exposure, likely reflecting the limited number of public epitopes available for HHV-7 and/or relatively poor representation of immunodominant linear epitopes for this virus in the array. More broadly, because the assay interrogates individual linear epitopes rather than full-length or conformational antigens, these measurements are expected to underrepresent true IgG seroprevalence across herpesviruses.^50^^,^^51^

In contrast to IgG, serum IgA responses to herpesvirus peptides were relatively sparse and highly heterogeneous (Fig. 4C). This pattern is consistent with the lower abundance of IgA in blood and with the fact that public epitope selection was largely informed by IgG-based serological mapping. Despite this overall sparsity, HHV-6 stood out with a disproportionate enrichment of IgA responses, particularly among individuals who developed LC. A subset of subjects exhibited high-amplitude IgA reactivity to multiple HHV-6 peptides. Relative to IgG, HHV-6 showed the strongest IgA signal on the array, indicating a virus- and isotype-specific response rather than a generalised increase in IgA.

To formally test whether high responders were enriched among individuals who developed LC, we applied an integrated upper-tail quantile analysis comparing the frequency and magnitude of responses in the upper tail of the distribution rather than shifts in central tendency. This analysis revealed virus- and isotype-specific enrichment of high responders in LC (Fig. 4B and D).

For IgG, the strongest and most consistent positive upper-tail shifts were observed among HHV-1 and HHV-2 epitopes, indicating that LC subjects were disproportionately represented among the highest IgG responders to these viruses across multiple tail cutoffs. Smaller but recurrent positive shifts were also observed for select HHV-4 (EBV) and HHV-5 (CMV) peptides, while other herpesviruses showed minimal or inconsistent IgG enrichment. These differences were mostly driven by enrichment among high responders rather than differences in median signal.

In contrast, IgA responses exhibited a distinct and more focused pattern. Upper-tail enrichment was concentrated almost exclusively among HHV-6 peptides, with multiple epitopes showing large positive integrated quantile differences across progressively stricter tail definitions (Fig. 4D). This pattern reflects both an increased frequency and increased magnitude of high IgA responders to HHV-6 in LC. Other herpesviruses showed little to no IgA tail enrichment. Scrambled control peptides showed no consistent directional shifts in either isotype, further supporting the specificity of these findings.

To visualise the distributions underlying these upper-tail effects, we examined background-subtracted MFI values for the top five peptides ranked by integrated quantile shift within each isotype (Fig. 4E and F). In both IgG and IgA, these peptides showed enrichment in LC+ subjects that was concentrated among a subset of extreme responders rather than reflecting a general shift across the full cohort. Thus, the observed group differences were driven primarily by the frequency and magnitude of upper-tail responses. Consistent with this interpretation, one-sided tail-focused Anderson-Darling permutation testing identified significant upper-tail enrichment for several peptides after Benjamini-Hochberg FDR correction.

### Herpesvirus-level isotype-specific associations with LC

Peptide-level signals were aggregated at the virus level using a peptide-normalised upper-tail framework to assess whether high-responder phenotypes clustered by herpesvirus species (Fig. 4G). This approach defined peptide-specific upper-tail events using pooled 90th percentile thresholds after a background-subtracted signal floor (MFI of 3000), and then quantified for each subject the percentage of peptides within each virus meeting those criteria. This reduced distortion from peptide-specific differences in dynamic range while emphasising subjects with more substantial upper-tail reactivity. For IgG, the largest positive differences were observed for HHV-1 and HHV-2. In contrast, IgA showed the strongest enrichment for HHV-6, with additional positive differences for HHV-4. Taken together, these data support the presence of isotype-specific herpesvirus antibody signatures in LC driven by an increased burden of upper-tail responders rather than a uniform increase in antiviral antibody levels across the cohort.

### Factorisation reveals LC-associated herpesvirus antibody modules

**Fig. 5:**
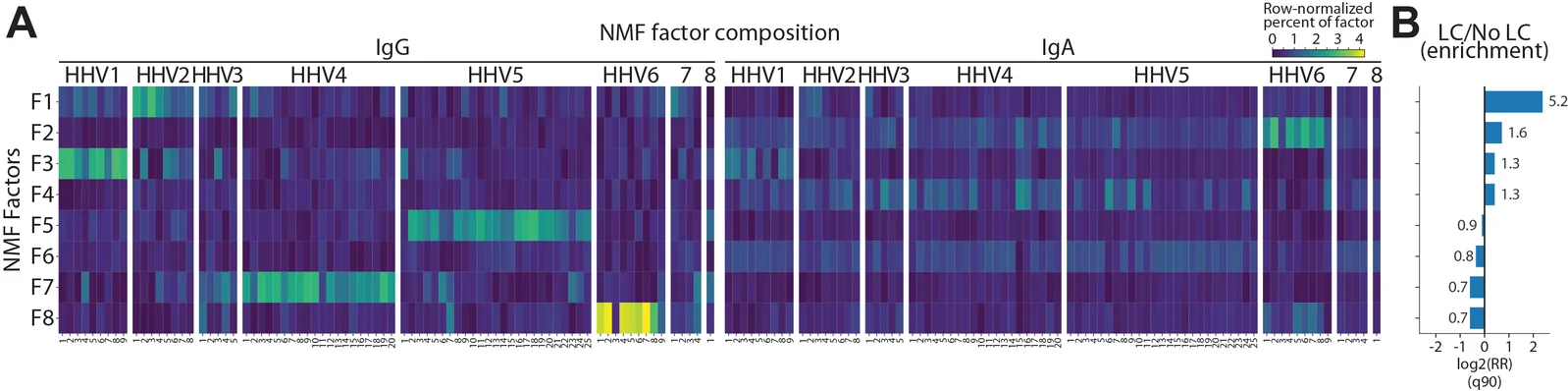
**NMF Factorisation of RECOVER cohort antibody profiles reveals modules differentially associated with LC.** Data are from the RECOVER analytic cohort (124 LC+ and 83 LC− participants). (A) Non-negative matrix factorisation of IgG and IgA epitope matrices reveals coherent antibody-response modules. Heat map shows row-normalised peptide loadings for each factor, grouped by viral species and isotype. (B) Association of non-negative matrix factorisation factor scores with long COVID. Bars represent log2 relative risk of LC+ status among subjects in the upper tail (≥90th percentile) of each factor score distribution, evaluated independently for each module.

To determine whether the peptide- and virus species–level associations observed above reflected broader, coordinated immune response patterns, we applied non-negative matrix factorisation (NMF) to dispersion-filtered IgG and IgA epitope matrices independent of LC status. This unsupervised approach identifies latent covariance structures—modules of peptides whose reactivity rises and falls together across individuals—agnostic to viral labels. The factorisation consistently recovered coherent and biologically interpretable modules, with peptides derived from the same herpesvirus tending to co-segregate within factors, thus providing strong internal validation of the assay and epitope selection (Fig. 5A). Specifically, several factors were dominated by IgG responses to individual herpesviruses, including HHV-1, HHV-2, HHV-4 (EBV), and HHV-5 (CMV), while others captured more restricted IgA-dominated responses. Notably, only HHV-6 exhibited a striking isotype bifurcation, forming one factor dominated by the IgA response and a separate factor dominated by the IgG response, in contrast to the correlated response between IgG and IgA observed for HHV-1 and HHV-2 factors. This pattern is surprising, as it indicates HHV-6 IgA and IgG responses are relatively uncoupled.

We next analysed the correlation of factor scores with clinical outcomes focused on log_2_ relative risk of LC among individuals in the upper tail (≥90th percentile). This revealed selective enrichment of LC among individuals with high scores in factors associated with HHV-1 IgG (Factor 3), HHV-2 IgG (Factor 1), HHV-6 IgA (Factor 2) and Factor 4 (constituting a less defined group of IgA epitopes to HHV-3, HHV-4 and HHV-5) (Fig. 5B). Other factors, including those associated with HHV-5 IgG, HHV-6 IgG and a more diffuse IgA pattern across HHV1-5 were inversely associated with LC. Overall, these factor-level associations mirrored the virus- and isotype-specific enrichment patterns identified by tail-based peptide MFI analyses, providing independent confirmation through an alternative analytical framework that LC is associated with isotype-specific herpesvirus antibody response modules rather than generalised antiviral activation.

### HHV-6 IgG associates with lower LC symptom burden

**Fig. 6:**
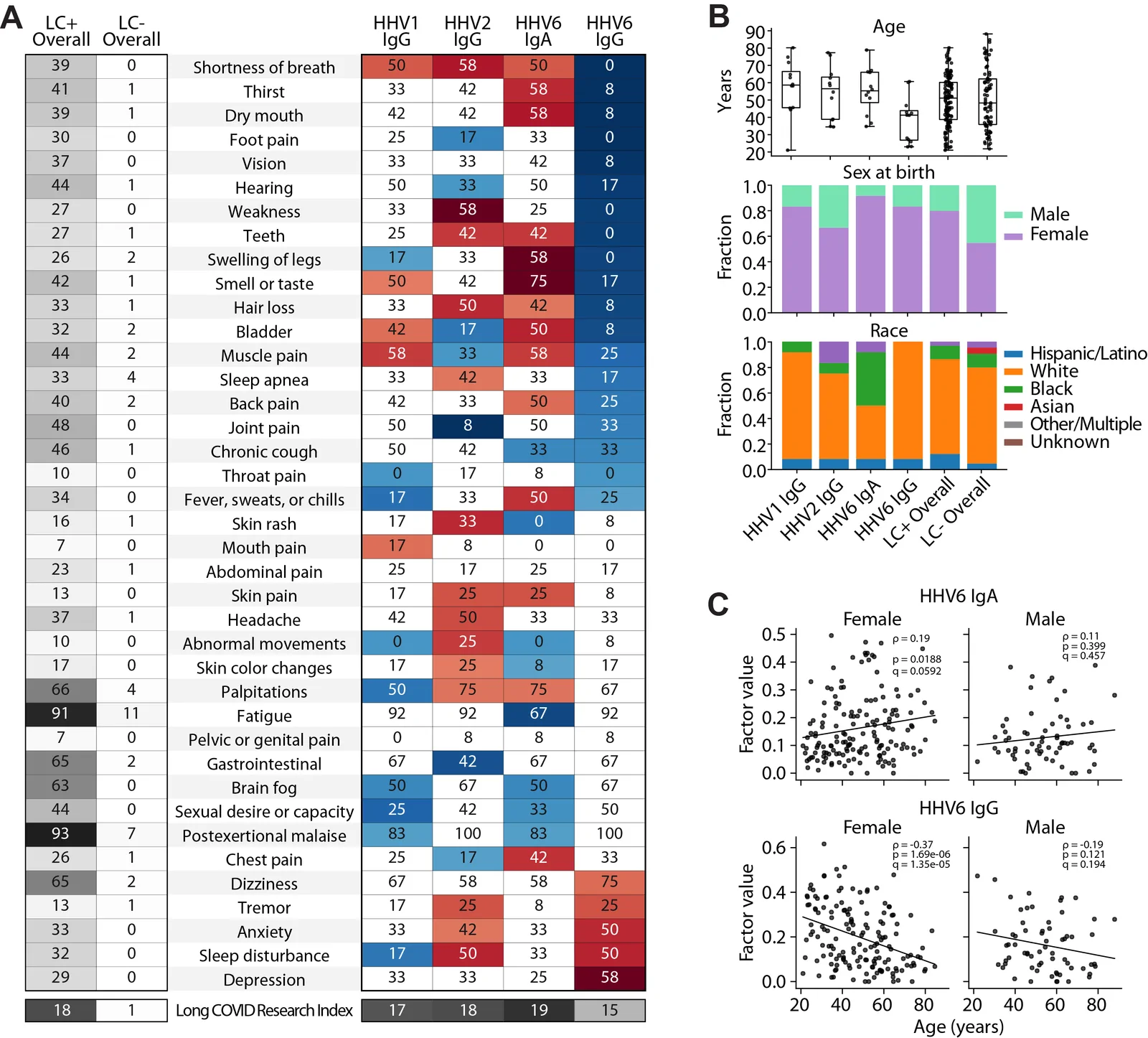
**Clinical and demographic correlates of herpesvirus antibody-response factors in RECOVER.** (A) On the left, the LC+ and LC− reference columns are shown as a greyscale, with darker shading indicating higher symptom prevalence. On the right, prevalence of long COVID symptoms among the top 12 individuals ranked by factor score for each indicated antibody-response module (HHV-1 IgG, HHV-2 IgG, HHV-6 IgA, and HHV-6 IgG). For antibody-response modules, heat-map colours indicate symptom prevalence relative to the overall LC+ group, with blue representing lower prevalence and red representing higher prevalence. Symptom categories are ordered by variance across groups. (B) Demographic characteristics of the top 12 individuals per factor compared with the overall LC+ and LC− groups. Each module-specific column represents the top 12 individuals (approximately the 90th percentile) ranked by factor score for that module; LC+ Overall and LC− Overall represent the full 124 and 83 participants, respectively, from the RECOVER analytic cohort. Box plots show age distributions; stacked bar plots show sex at birth and self-reported race/ethnicity. (C) Association between continuous factor scores and age across all analysed subjects. Scatter plots show factor values as a function of age for HHV-6 IgA and HHV-6 IgG modules, stratified by sex at birth, with fitted trend lines. Spearman correlation coefficients (*ρ*), nominal *P* values, and Benjamini-Hochberg-adjusted *q* values are shown.

To examine how herpesvirus antibody response factors relate to clinical features of long COVID, we analysed the factors associated with altered LC risk, including HHV-1 IgG, HHV-2 IgG, HHV-6 IgG, and HHV-6 IgA (Fig. 6). For each factor, we focused on the 12 individuals with the highest scores, representing those in whom the corresponding antibody program was most prominent. Using symptom definitions and LC classification based on the revised Long COVID Research Index (LCRI) published by Geng et al., overall symptom prevalence in the RECOVER subset analysed here was similar to the reference cohort (Fig. 6A).^35^ When stratified by antibody-defined subgroups, individuals enriched for HHV-1 IgG, HHV-2 IgG, or HHV-6 IgA modules showed similar to slightly increased overall symptom burden compared with the overall LC+ group, but without clearly distinct module-associated symptom features. Thus, the herpesvirus antibody programs most strongly associated with LC identified individuals with broadly elevated symptom burdens rather than clearly distinct clinical phenotypes. In contrast, individuals with high HHV-6 IgG factor scores displayed a distinct clinical profile characterised by lower prevalence of multiple systemic LC-associated symptoms and increased representation among participants classified as LC−. Furthermore, the mean LCRI for each group reflected these observations, with the lowest mean LCRI present in the HHV-6 IgG group (Fig. 6A, bottom row). Notably, this relative reduction in systemic symptom burden did not extend uniformly across all symptom domains, and neuropsychiatric symptoms appeared relatively preserved or modestly enriched in this subgroup. This pattern parallels the neutral or inverse association of the HHV-6 IgG module with LC observed in factor-level enrichment analyses and is consistent with HHV-6-directed IgA- and IgG-dominated antibody programs representing biologically and clinically distinct immune states.

### Age-related decline in HHV-6 IgG responses

Demographic characteristics of the top 12 individuals per factor and the overall LC+ and LC− groups are summarised in Fig. 6B. While age distributions overlapped across factors, the HHV-6 IgG group showed a lower median age, decreasing from approximately 55 years in the HHV-1 IgG, HHV-2 IgG, and HHV-6 IgA groups to approximately 41 years in the HHV-6 IgG group. Sex-at-birth distributions differed modestly, with female participants comprising a larger fraction of the HHV-6 IgG high-responder group. Racial and ethnic composition largely reflected the underlying cohort and did not show strong factor-specific enrichment.

To determine whether the association of age with factor expression levels extended beyond extreme responders, we examined this across the full RECOVER cohort independent of LC status (Fig. 6C). Across the full cohort, HHV-6 IgA factor scores showed a minor positive correlation with age. In contrast, HHV-6 IgG factor scores exhibited a significant inverse relationship with age, evident in both sexes; the association reached statistical significance only among females, likely reflecting the larger female sample size in the cohort. No comparable age associations were observed for HHV-1 IgG or HHV-2 IgG factor scores (Supplementary Fig. S2). Thus, the opposing age dependencies of HHV-6 IgA and IgG antibody responses appear to reflect virus-specific, rather than generalised effects of age or antibody magnitude.

## Discussion

In this study, we developed and applied a minimal public epitope, peptide-based microbead platform to enable epitope-resolved profiling of antibody responses across all eight human herpesviruses. Using this approach in longitudinal acute-infection cohorts and early post-infection samples from the NIH RECOVER initiative, we identified structured herpesvirus antibody signatures that emerge during acute SARS-CoV-2 infection and associate with subsequent development of LC. LC was characterised by enrichment of high responders in a virus- and isotype-specific manner, most prominently IgG enrichment for HHV-1 and HHV-2 and IgA enrichment for HHV-6. Unsupervised factorisation cleanly separated HHV-6 IgA- and HHV-6 IgG-dominated modules, which exhibited opposing clinical and demographic associations: HHV-6 IgA responses were linked to higher LC symptom burden and increased with age, whereas HHV-6 IgG responses declined with age and were associated with lower symptom prevalence and enrichment among participants without LC. Together, these findings support a model in which LC risk is linked to isotype-specific herpesvirus antibody programs expressed in subsets of individuals early during acute SARS-CoV-2 infection.

Proposed mechanisms of LC include persistent immune activation, dysregulated innate and adaptive immunity, autoantibody production, endothelial and microvascular injury, complement activation, and neuroendocrine perturbations.^2^^,^^3^^,^^11^^,^^14^^,^^18^ Furthermore, existing evidence supports a model in which early divergence in inflammatory markers, viral and immune features, and antibody responses differentiates individuals who later develop LC.^2^^,^^11^, ^12^, ^13^^,^^19^ In this context, the isotype-restricted surge of herpesvirus-directed IgA we observed—particularly against beta-herpesviruses, and without parallel increases in IgG or IgM—suggests a pattern of mucosal immune engagement rather than a coordinated systemic response. This is potentially consistent with localised viral antigen exposure and/or bystander immune activation near mucosal sites of primary SARS-CoV-2 infection.

Herpesvirus reactivation, particularly Epstein–Barr virus (EBV/HHV-4), has been repeatedly proposed as a contributor to LC pathogenesis, with mixed evidence across several studies reporting associations between LC and EBV serologic markers or other markers of recent viral activity.^13^^,^^15^^,^^16^^,^^20^^,^^22^ Reactivation of other herpesviruses, namely HSV-1, CMV, HHV-6 and HHV-7, has been observed during acute SARS-CoV-2 by viral DNA detection in blood.^22^^,^^52^, ^53^, ^54^, ^55^ With regard to HHV-6 specifically, emerging associative studies have reported serologic signals in LC.^56^ By contrast, direct evidence of HHV-6 DNA during acute COVID has been described only in a subset of patients.^53^, ^54^, ^55^ These findings remain poorly understood with regard to HHV-6 biology and difficult to interpret with respect to timing, reactivation, and mechanism in LC.^56^^,^^57^

The IgA-dominant, epitope-resolved antibody signatures we report here differ substantially from prior studies. Our findings reveal early IgA-focused antibody patterns driven by high-responder subsets directed against specific epitopes that are largely undetectable using conventional serologic approaches. Notably, all but one of the HHV-6 public epitopes in our panel were derived from the U47 glycoprotein, a late lytic marker. U47 was selected due to the high representation of public epitopes derived from this protein. Therefore, this screen does not capture the full antigenic breadth of HHV-6. Nevertheless, the U47 epitopes were responsible for our observed LC associations, highlighting the value of epitope-resolved profiling for identifying selective immune programs that are obscured in conventional serologic assays.^31^, ^32^, ^33^, ^34^ Overall, these findings support the possibility that the observed HHV-6 antibody signatures reflect mucosal HHV-6 antigen exposure, bystander immune activation, or other nonclassical forms of herpesvirus immune engagement.

We observed that herpesvirus antibody signals associated with LC were driven predominantly by high responders to specific viral epitopes. This pattern suggests a subset of individuals may enter distinct immune states marked by bystander activation, barrier-associated immune engagement, localised antigen exposure, or selective re-engagement of pre-existing memory B cell clones.^28^^,^^29^^,^^58^ Similar patterns have been described in other immune-mediated diseases. For example, stronger EBV serologic responses, including higher VCA IgG and EA IgG levels, have been associated with transition to systemic lupus erythematosus in at-risk relatives, suggesting biologically meaningful disease-associated immune states.^59^^,^^60^ HHV-6–directed immune responses have likewise been associated with conversion or relapse risk in multiple sclerosis.^61^ Viewed in this broader context, the high-responder structure observed here is consistent with biologically distinct subgroups or immune states that may be associated with susceptibility to LC.

HHV-6 antibody responses exhibited particularly striking structure. Factorisation resolved HHV-6-directed responses into distinct IgA- and IgG-dominated modules with minimal cross-loading, and these modules showed opposing clinical and demographic associations. HHV-6 IgA was associated with greater LC symptom burden and remained stable or increased slightly with age, whereas HHV-6 IgG was associated with lower symptom burden, relative enrichment among participants without LC, and declining levels with age. By contrast, the HHV-1 and HHV-2 signals associated with LC were observed primarily in IgG, with partial covariance in IgA, suggesting a different immune architecture than the marked isotype divergence seen for HHV-6. Thus, although HHV-1 and HHV-2 remain relevant to LC biology, the opposing IgA and IgG associations within HHV-6 suggest a more differentiated immune architecture in which the two isotypes do not simply track as parallel measures of the same antiviral response.

One interpretation of the differing HHV-6 IgA and IgG patterns associated with ageing is that later-life HHV-6-directed IgA reflects ongoing barrier-associated immune engagement that is not parallelled by maintenance of systemic IgG responses, consistent with the broader role of IgA in mucosal antimicrobial defence.^28^ Enrichment of HHV-6-directed IgA—particularly alongside lower HHV-6 IgG—may mark an immune state associated with increased susceptibility to downstream LC pathophysiology, whereas stronger systemic humoural control may be associated with lower risk. This is biologically plausible because HHV-6 establishes lifelong persistence after early-life acquisition and is frequently detected in saliva and salivary compartments, consistent with mucosal shedding and compartment-specific viral activity.^57^^,^^62^ Acute SARS-CoV-2 infection, which begins at mucosal surfaces and induces partly compartmentalised local and systemic immune responses during acute infection, could therefore create an inflammatory environment for localised HHV-6 antigen exposure or shedding without necessarily producing a parallel systemic serologic pattern.^24^^,^^26^^,^^29^^,^^48^

This study has several limitations. Pre-infection baseline samples were not available for the early RECOVER cohort, precluding definitive distinction between pre-existing antibody levels and infection-triggered changes, although longitudinal acute-infection data support a SARS-CoV-2-associated rise in herpesvirus-directed IgA. The longitudinal acute-infection cohort was also relatively small (n = 12), limiting statistical power and the precision and generalisability of the observed temporal antibody trajectories. In addition, linear peptide arrays do not capture conformational epitopes or directly establish how reactivity to the measured peptides relates to antibody responses against full, folded viral proteins, particularly for IgG responses in antiviral immunity.^50^^,^^51^ Peptide quality represents a further limitation. Although all peptides underwent HPLC-based quality-control review and exhibited a dominant product-associated elution region, several chromatograms showed secondary peaks, peak splitting, shoulders, or broad unresolved elution profiles. Consequently, measured antibody reactivity cannot be attributed with complete certainty to a single chemical species for every peptide and may partially reflect binding to closely related synthesis by-products. However, the reproducibility of the principal findings across independent cohorts, multiple analytical frameworks, and groups of biologically related peptides argues against the major conclusions being driven by individual peptide impurities. At the same time, this approach enables measurement of responses to individual peptide epitopes, providing resolution that is not available from whole-protein or lysate-based serologic assays. Age represents an additional potential confounder, as increasing age is itself associated with elevated long COVID risk and could contribute to the observed age-dependent shifts in HHV-6-directed antibody responses, generating associations with long COVID without implying direct causality.^4^^,^^7^ The stronger age-associated trends observed in females should be interpreted cautiously, as the RECOVER cohort contained substantially more female than male participants. The smaller male subgroup therefore had reduced statistical power to detect associations of comparable magnitude. However, the opposing age dependencies observed for HHV-6 IgA and HHV-6 IgG—together with their divergent clinical associations—argue against age acting as a simple global driver of antibody magnitude and are instead consistent with biologically distinct, isotype-specific immune programs. Finally, tail-based enrichment analyses rely on smaller effective sample sizes than population-wide analyses, although this limitation was mitigated through integrated quantile approaches and conservative upper-tail definitions.

Future studies should include replication in independent early-infection cohorts with prospective LC follow-up, direct comparison with other early LC risk markers, and integration of orthogonal measures of herpesvirus activity. For HHV-6 studies, pairing serum and mucosal samples will be critical to determine whether the HHV-6 IgA signature reflects mucosal spillover, localised antigen exposure, or systemic induction.^24^^,^^28^^,^^48^ In addition, the performance, reproducibility, and predictive value of compact, isotype-resolved, peptide epitope antibody signatures will need to be rigorously tested across diverse clinical settings before their reproducibility and biological relevance can be established. If validated, such signatures could provide a practical framework for identifying individuals at elevated risk for LC, although such applications remain investigational.

## Contributors

A.C., P.J.U., and T.R.P. designed experiments and peptide array panels. A.C., T.R.P., M.J.B., W.J.K., X.Y., M.H., A.R., and M.K. generated antigen arrays. A.C., T.R.P., W.J.K., S.P., and M.J.B. prepared clinical samples for analysis. A.C., M.J.B., and T.R.P. performed all sample runs, quality control, and data processing. A.C., M.J.B., A.R., M.K., and M.H. performed additional autoantibody screening arrays and interpreted the related data. G.S.N. and E.Q.C. performed qPCR assay development and analysis. J.F. provided healthy control samples crucial for assay development. E.W.K. and J.B. assisted with sample coordination and distribution. A.C., T.R.P., and P.J.U. analysed and interpreted data and generated figures. A.C., T.R.P., and P.J.U. wrote the manuscript. A.C., T.R.P., and P.J.U. accessed and verified the underlying data. All authors have read and approved the final version of the manuscript.

## Data sharing statement

The processed data supporting the findings of this study have been deposited in Zenodo (record 20821345) and is publicly available. Requests for the analysis code should be directed to the corresponding author at tyler4@stanford.edu. RECOVER clinical data and biospecimens are governed by NIH RECOVER data access policies and are therefore not redistributed by the authors; qualified investigators may obtain access through the NIH RECOVER program. All other data supporting the findings of this study are available within the manuscript, its Supplementary Information, or the deposited Zenodo repository.

## Declaration of interests

The authors declare no competing interests.

## Appendix A Supplementary data

Supplementary Figures

Supplementary Protocol

Reagent Validation
