# Supplementary material for: Early HHV-6 IgG and IgA responses during acute SARS-CoV-2 infection in relation to long COVID: a multicohort observational study
Source: eBioMedicine. 2026 Aug 27;131:106455. doi: 10.1016/j.ebiom.2026.106455 (PMC13544345; doi:10.1016/j.ebiom.2026.106455)
Supplement: Supplementary file 1 [file mmc1.docx]

### **SUPPLEMENTARY TABLES**

### **Supplementary Table S1. Overview of cohorts included in this study**

| **Cohort** | **Purpose in current study** | **Participant population** | **Sampling timepoint** | **Samples included in current analysis** | **Key outcome/**  **phenotype** |
| --- | --- | --- | --- | --- | --- |
| CHART/  Track Covid | Longitudinal acute infection profiling | Community- based Adults/Hospital Employees with confirmed SARS-CoV-2 infection | Serial samples collected during acute infection and early recovery | n = 12 participants | Temporal herpesvirus antibody responses during acute COVID-19 |
| NIH RECOVER | Early post-infection long COVID analysis | Adults enrolled in RECOVER observational study | Samples collected within 30 days of SARS-CoV-2 infection | n = 207 participants (124 LC+, 83 LC−) | Long COVID status defined using Long COVID Research Index (LCRI) |

CHART = COVID-19 Host Immune Response Pathogenesis Program. RECOVER = Researching COVID to Enhance Recovery. Long COVID status was defined using the Long COVID Research Index (LCRI), with participants classified as LC+ (LCRI ≥11) or LC− (LCRI 0–10).

# **Supplementary Table S2. Herpesvirus peptide panel used for epitope-resolved antibody profiling**

| **ID** | **Virscan ID** | **Organism** | **Protein names** | **Minimal public epitope sequence** |
| --- | --- | --- | --- | --- |
| HHV1 1 | 113898 | Human herpesvirus 1 (HHV-1) (Human herpes simplex virus 1) | Glycoprotein G (Fragment) | EEEEEEGAGDGEHLE |
| HHV1 2 | 113899 | Human herpesvirus 1 (HHV-1) (Human herpes simplex virus 1) | Glycoprotein G (Fragment) | GDGTRDTLPQSPGPAFPLAEDVEKDKPNRP |
| HHV1 3 | 41949 | Human herpesvirus 1 (strain 17) (HHV-1) (Human herpes simplex virus 1) | Capsid scaffolding protein (Capsid protein P40) (Protease precursor) (pPR) (Virion structural protein UL26) [Cleaved into: Assemblin (EC 3.4.21.97) (Capsid protein VP24) (Protease); Assembly protein (Capsid protein VP22A)] | ARAADLFVSQMMGAR |
| HHV1 4 | 42521 | Human herpesvirus 1 (strain 17) (HHV-1) (Human herpes simplex virus 1) | Deneddylase (EC 3.4.19.12) (EC 3.4.22.-) (Tegument protein VP1-2) (Tegument protein VP1/2) | PQPQPQPQPQPQPQPQPQPQPQPQP |
| HHV1 5 | 98559 | Human herpesvirus 1 (strain 17) (HHV-1) (Human herpes simplex virus 1) | Envelope glycoprotein D (gD) | RRHTQKAPKRIRLPH |
| HHV1 6 | 27834 | Human herpesvirus 1 (strain 17) (HHV-1) (Human herpes simplex virus 1) | Envelope glycoprotein G (gG) (gG-1) | EKDKPNRPVVPSPDP |
| HHV1 7 | 22973 | Human herpesvirus 1 (strain 17) (HHV-1) (Human herpes simplex virus 1) | Envelope glycoprotein M (gM) | VGGYDPEPAEDPVYSTVRRW |
| HHV1 8 | 120092 | Human herpesvirus 1 (strain Patton) (HHV-1) (Human herpes simplex virus 1) | Envelope glycoprotein B (gB) (gB-1) (gB1) | PALGAAPTGDPKPKKNKKPK |
| HHV1 9 | 120091 | Human herpesvirus 1 (strain Patton) (HHV-1) (Human herpes simplex virus 1) | Envelope glycoprotein B (gB) (gB-1) (gB1) | NKKPKNPTPPRPAGD |
| HHV2 1 | 116946 | Human herpesvirus 2 (HHV-2) (Human herpes simplex virus 2) | Glycoprotein G-2 (Fragment) | RGGPEEFEGAGDGEP |
| HHV2 2 | 14022 | Human herpesvirus 2 (strain 333) (HHV-2) (Human herpes simplex virus 2) | Envelope glycoprotein D (gD) | RRAQMAPKRLRLPHI |
| HHV2 3 | 121425 | Human herpesvirus 2 (strain B4327UR) (HHV-2) (Human herpes simplex virus 2) | Envelope glycoprotein G (gG) (gG-2) | PEHRGGPEEFEGAGDGEPPE |
| HHV2 4 | 121426 | Human herpesvirus 2 (strain B4327UR) (HHV-2) (Human herpes simplex virus 2) | Envelope glycoprotein G (gG) (gG-2) | PKTHPHGPADAPPGS |
| HHV2 5 | 75123 | Human herpesvirus 2 (strain HG52) (HHV-2) (Human herpes simplex virus 2) | Capsid scaffolding protein (Capsid protein P40) (Protease precursor) (pPR) (Virion structural protein UL26) [Cleaved into: Assemblin (EC 3.4.21.97) (Capsid protein VP24) (Protease); Assembly protein (Capsid protein VP22A)] | ARAADLFVSQMMGSR |
| HHV2 6 | 74647 | Human herpesvirus 2 (strain HG52) (HHV-2) (Human herpes simplex virus 2) | Envelope glycoprotein M (gM) | SVSHHAEIDRYGDSDGDPIYDEVAP |
| HHV2 7 | 75410 | Human herpesvirus 2 (strain HG52) (HHV-2) (Human herpes simplex virus 2) | Envelope protein US9 (10 kDa protein) | EDEAANDFLVRMGRQQSVLR |
| HHV2 8 | 59003 | Herpes simplex virus type 2 (strain SA8) (Simian agent 8) | Envelope glycoprotein B (gB) (gB-2) (gB2) | HKARKKGTSALLSAK |
| HHV3 1 | 104085 | Human herpesvirus 3 (HHV-3) (Varicella-zoster virus) | Tegument protein | PVQPAQPVQPAQPAQTVQPAQPAQPVQPAQPAQPA |
| HHV3 2 | 39662 | Varicella-zoster virus (strain Dumas) (HHV-3) (Human herpesvirus 3) | Capsid protein VP26 homolog | KGQTLSHTGQSGNASRSRRV |
| HHV3 3 | 38234 | Varicella-zoster virus (strain Dumas) (HHV-3) (Human herpesvirus 3) | Envelope glycoprotein B (gB) (Glycoprotein II) | EMIKYMTLVSAAERQESKAR |
| HHV3 4 | 37187 | Varicella-zoster virus (strain Dumas) (HHV-3) (Human herpesvirus 3) | Envelope glycoprotein C (gC) (Glycoprotein V) (gpV) | SRKPDPAVAPTSAATRKPDPAVAPTSAASR |
| HHV3 5 | 39376 | Varicella-zoster virus (strain Dumas) (HHV-3) (Human herpesvirus 3) | Tegument protein VP22 (ORF9 protein) (Tegument protein 9) | EPMYAQVRKPKSRTD |
| HHV4 1 | 72154 | Epstein-Barr virus (strain AG876) (HHV-4) (Human herpesvirus 4) | Envelope glycoprotein GP350 (Membrane antigen) (MA) | SKLRPRWTFTSPPVT |
| HHV4 10 | 51285 | Epstein-Barr virus (strain B95-8) (HHV-4) (Human herpesvirus 4) | Capsid protein VP26 | GGQPHDTAPRGARKKQ |
| HHV4 11 | 50144 | Epstein-Barr virus (strain B95-8) (HHV-4) (Human herpesvirus 4) | Capsid protein VP26 | QQAQAAASAGTGALASSAPS |
| HHV4 12 | 14879 | Epstein-Barr virus (strain B95-8) (HHV-4) (Human herpesvirus 4) | Epstein-Barr nuclear antigen 4 (EBNA-4) (EBV nuclear antigen 4) (Epstein-Barr nuclear antigen 3B) (EBNA-3B) (EBV nuclear antigen 3B) | EDPPDYGDQGNVTQVGSEPISPEIGPFELS |
| HHV4 13 | 14878 | Epstein-Barr virus (strain B95-8) (HHV-4) (Human herpesvirus 4) | Epstein-Barr nuclear antigen 4 (EBNA-4) (EBV nuclear antigen 4) (Epstein-Barr nuclear antigen 3B) (EBNA-3B) (EBV nuclear antigen 3B) | QADAGGASGSEDPPDYGDQGNVTQV |
| HHV4 14 | 99132 | Epstein-Barr virus (strain GD1) (HHV-4) (Human herpesvirus 4) | BZLF1 | MMDPNSTSEDVKFTP |
| HHV4 15 | 99133 | Epstein-Barr virus (strain GD1) (HHV-4) (Human herpesvirus 4) | BZLF1 | PFVQAFDQATRVYQDLGGPS |
| HHV4 16 | 99131 | Epstein-Barr virus (strain GD1) (HHV-4) (Human herpesvirus 4) | BZLF1 | DPYQVPFVQAFDQAT |
| HHV4 17 | 90461 | Epstein-Barr virus (strain GD1) (HHV-4) (Human herpesvirus 4) | Epstein-Barr nuclear antigen 2 (EBNA-2) (EBV nuclear antigen 2) | LPPTPLLTVLQRPTE |
| HHV4 18 | 90080 | Epstein-Barr virus (strain GD1) (HHV-4) (Human herpesvirus 4) | Epstein-Barr nuclear antigen 6 (EBNA-6) (EBV nuclear antigen 6) (Epstein-Barr nuclear antigen 3C) (EBNA-3C) (EBV nuclear antigen 3C) (Epstein-Barr nuclear antigen 4B) (EBNA-4B) (EBV nuclear antigen 4B) | YQGYQEQPAPQAPYQGYQEP |
| HHV4 19 | 97702 | Epstein-Barr virus (strain GD1) (HHV-4) (Human herpesvirus 4) | Epstein-Barr virus nuclear antigen (EBNA2) (Fragment) | SPEAPILFPDDWYPPSIDPADLDES |
| HHV4 2 | 80840 | Epstein-Barr virus (strain AG876) (HHV-4) (Human herpesvirus 4) | Epstein-Barr nuclear antigen 1 (EBNA-1) (EBV nuclear antigen 1) | GDDGDEGGDGDEGEE |
| HHV4 20 | 97701 | Epstein-Barr virus (strain GD1) (HHV-4) (Human herpesvirus 4) | Epstein-Barr virus nuclear antigen (EBNA2) (Fragment) | PESHNSPEAPILFPDDWYPP |
| HHV4 3 | 80841 | Epstein-Barr virus (strain AG876) (HHV-4) (Human herpesvirus 4) | Epstein-Barr nuclear antigen 1 (EBNA-1) (EBV nuclear antigen 1) | PPWFPPMVEGAAAEGDDGDDGDEGGDGDEGEEGQE |
| HHV4 4 | 79317 | Epstein-Barr virus (strain AG876) (HHV-4) (Human herpesvirus 4) | Epstein-Barr nuclear antigen 1 (EBNA-1) (EBV nuclear antigen 1) | RDGVRRPQKRPSCIGCKGAHGGTGA |
| HHV4 5 | 80079 | Epstein-Barr virus (strain AG876) (HHV-4) (Human herpesvirus 4) | Epstein-Barr nuclear antigen 1 (EBNA-1) (EBV nuclear antigen 1) | PPPGRRPFFHPVAEADYFEYHQEGG |
| HHV4 6 | 82460 | Epstein-Barr virus (strain AG876) (HHV-4) (Human herpesvirus 4) | Epstein-Barr nuclear antigen leader protein (EBNA-LP) (EBV nuclear antigen leader protein) (Epstein-Barr nuclear antigen 5) (EBNA-5) (EBV nuclear antigen 5) | PGIGPEGPLGQLLRRHRSPS |
| HHV4 7 | 81792 | Epstein-Barr virus (strain AG876) (HHV-4) (Human herpesvirus 4) | Tegument protein BRRF2 | DVQREFSGLRVIGDEDEDGS |
| HHV4 8 | 81793 | Epstein-Barr virus (strain AG876) (HHV-4) (Human herpesvirus 4) | Tegument protein BRRF2 | SKKGADEFEAWLEAQ |
| HHV4 9 | 15925 | Epstein-Barr virus (strain AG876) (HHV-4) (Human herpesvirus 4) | Tegument protein UL11 homolog | GETNEGLEYDEDSENDELLFLPNKK |
| HHV5 1 | 100370 | Human cytomegalovirus (HHV-5) (Human herpesvirus 5) | Glycoprotein gp58/116 (Fragment) | SHATSSAHNGSHTSR |
| HHV5 10 | 33280 | Human cytomegalovirus (strain AD169) (HHV-5) (Human herpesvirus 5) | Large structural phosphoprotein (150 kDa matrix phosphoprotein) (150 kDa phosphoprotein) (pp150) (Basic phosphoprotein) (BPP) (Phosphoprotein UL32) (Tegument protein UL32) | GNSPWAPTAPLPGDM |
| HHV5 11 | 33281 | Human cytomegalovirus (strain AD169) (HHV-5) (Human herpesvirus 5) | Large structural phosphoprotein (150 kDa matrix phosphoprotein) (150 kDa phosphoprotein) (pp150) (Basic phosphoprotein) (BPP) (Phosphoprotein UL32) (Tegument protein UL32) | PRERAWALKNPHLAYNPFRM |
| HHV5 12 | 31946 | Human cytomegalovirus (strain AD169) (HHV-5) (Human herpesvirus 5) | Large structural phosphoprotein (150 kDa matrix phosphoprotein) (150 kDa phosphoprotein) (pp150) (Basic phosphoprotein) (BPP) (Phosphoprotein UL32) (Tegument protein UL32) | PARKPSASRRLFGSSADEDD |
| HHV5 13 | 36710 | Human cytomegalovirus (strain AD169) (HHV-5) (Human herpesvirus 5) | Large structural phosphoprotein (150 kDa matrix phosphoprotein) (150 kDa phosphoprotein) (pp150) (Basic phosphoprotein) (BPP) (Phosphoprotein UL32) (Tegument protein UL32) | PQKSGTGPQPGSAGMGGAKTPSDAV |
| HHV5 14 | 32614 | Human cytomegalovirus (strain AD169) (HHV-5) (Human herpesvirus 5) | Large structural phosphoprotein (150 kDa matrix phosphoprotein) (150 kDa phosphoprotein) (pp150) (Basic phosphoprotein) (BPP) (Phosphoprotein UL32) (Tegument protein UL32) | QQQQRHAAFSLVSPQVTKAS |
| HHV5 15 | 32613 | Human cytomegalovirus (strain AD169) (HHV-5) (Human herpesvirus 5) | Large structural phosphoprotein (150 kDa matrix phosphoprotein) (150 kDa phosphoprotein) (pp150) (Basic phosphoprotein) (BPP) (Phosphoprotein UL32) (Tegument protein UL32) | RHAAFSLVSPQVTKA |
| HHV5 16 | 34519 | Human cytomegalovirus (strain AD169) (HHV-5) (Human herpesvirus 5) | Large structural phosphoprotein (150 kDa matrix phosphoprotein) (150 kDa phosphoprotein) (pp150) (Basic phosphoprotein) (BPP) (Phosphoprotein UL32) (Tegument protein UL32) | VLGKRVATPHASARA |
| HHV5 17 | 35949 | Human cytomegalovirus (strain AD169) (HHV-5) (Human herpesvirus 5) | Large structural phosphoprotein (150 kDa matrix phosphoprotein) (150 kDa phosphoprotein) (pp150) (Basic phosphoprotein) (BPP) (Phosphoprotein UL32) (Tegument protein UL32) | KSSVSNAPPVASPSILKPGASAALQ |
| HHV5 18 | 35092 | Human cytomegalovirus (strain AD169) (HHV-5) (Human herpesvirus 5) | Large structural phosphoprotein (150 kDa matrix phosphoprotein) (150 kDa phosphoprotein) (pp150) (Basic phosphoprotein) (BPP) (Phosphoprotein UL32) (Tegument protein UL32) | PSLKPTLGGKAVVGRPPSVPVSGSA |
| HHV5 19 | 46902 | Human cytomegalovirus (strain AD169) (HHV-5) (Human herpesvirus 5) | Tegument protein UL11 homolog (28 kDa structural phosphoprotein) (pp28) | DLDEEDTSIYLSPPPVPPVQ |
| HHV5 2 | 100369 | Human cytomegalovirus (HHV-5) (Human herpesvirus 5) | Glycoprotein gp58/116 (Fragment) | SSEAVSHRANETIYNTTLKY |
| HHV5 20 | 46904 | Human cytomegalovirus (strain AD169) (HHV-5) (Human herpesvirus 5) | Tegument protein UL11 homolog (28 kDa structural phosphoprotein) (pp28) | KKKPSKHHHHQQSSI |
| HHV5 21 | 46903 | Human cytomegalovirus (strain AD169) (HHV-5) (Human herpesvirus 5) | Tegument protein UL11 homolog (28 kDa structural phosphoprotein) (pp28) | PPVQVVAKRLPRPDTPRTPR |
| HHV5 22 | 52714 | Human cytomegalovirus (strain AD169) (HHV-5) (Human herpesvirus 5) | Tegument protein UL71 | VKKKKPTAAALLSSA |
| HHV5 23 | 47950 | Human cytomegalovirus (strain Towne) (HHV-5) (Human herpesvirus 5) | Envelope glycoprotein B (gB) [Cleaved into: Glycoprotein GP55] | ASTAAPPYTNEQAYQMLLAL |
| HHV5 24 | 47951 | Human cytomegalovirus (strain Towne) (HHV-5) (Human herpesvirus 5) | Envelope glycoprotein B (gB) [Cleaved into: Glycoprotein GP55] | EQAYQMLLALVRLDAEQRAQ |
| HHV5 25 | 49285 | Human cytomegalovirus (strain Towne) (HHV-5) (Human herpesvirus 5) | Envelope glycoprotein B (gB) [Cleaved into: Glycoprotein GP55] | LLDRLRHRKNGYRHL |
| HHV5 3 | 109229 | Human cytomegalovirus (HHV-5) (Human herpesvirus 5) | Matrix phosphoprotein (Fragment) | DEVWALRDQTAESPVEDSEE |
| HHV5 4 | 147358 | Human cytomegalovirus (HHV-5) (Human herpesvirus 5) | Membrane glycoprotein UL139 | KTTHAYTNAAFTSSDATLPM |
| HHV5 5 | 147357 | Human cytomegalovirus (HHV-5) (Human herpesvirus 5) | Membrane glycoprotein UL139 | MGTTGSYTPPQDGSFPPPPR |
| HHV5 6 | 2761 | Human cytomegalovirus (HHV-5) (Human herpesvirus 5) | UL132 protein (Fragment) | NEEDPIRYYVSVYDELTASE |
| HHV5 7 | 2763 | Human cytomegalovirus (HHV-5) (Human herpesvirus 5) | UL132 protein (Fragment) | NSTSWQIPKLMKVATQPVSL |
| HHV5 8 | 51858 | Human cytomegalovirus (strain AD169) (HHV-5) (Human herpesvirus 5) | Envelope glycoprotein M (gM) | HHDSLESRRLREEEDDDDDEDFEDA |
| HHV5 9 | 31947 | Human cytomegalovirus (strain AD169) (HHV-5) (Human herpesvirus 5) | Large structural phosphoprotein (150 kDa matrix phosphoprotein) (150 kDa phosphoprotein) (pp150) (Basic phosphoprotein) (BPP) (Phosphoprotein UL32) (Tegument protein UL32) | DDDDEKNIFTPIKKP |
| HHV6 1 | 63577 | Human herpesvirus 6A (strain GS) (HHV-6 variant A) (Human B lymphotropic virus) | Glycoprotein U47 | PKNSIYLKSKSQQSTTKFTD |
| HHV6 2 | 62911 | Human herpesvirus 6A (strain GS) (HHV-6 variant A) (Human B lymphotropic virus) | Glycoprotein U47 | KKIVKRSDFPRPMYTPTDIP |
| HHV6 3 | 101704 | Human herpesvirus 6A (strain Uganda-1102) (HHV-6 variant A) (Human B lymphotropic virus) | U87 | QAKPPVKPQAKPPAEPPVGI |
| HHV6 4 | 69011 | Human herpesvirus 6B (strain Z29) (HHV-6 variant B) (Human B lymphotropic virus) | Glycoprotein U47 | KVVKRSDFPRPMYTP |
| HHV6 5 | 69582 | Human herpesvirus 6B (strain Z29) (HHV-6 variant B) (Human B lymphotropic virus) | Glycoprotein U47 | SDNIYSSPKNSIYLKSKSQQ |
| HHV6 6 | 69963 | Human herpesvirus 6B (strain Z29) (HHV-6 variant B) (Human B lymphotropic virus) | Glycoprotein U47 | STEVQTVKTFKEDRFQRTTL |
| HHV6 7 | 70725 | Human herpesvirus 6B (strain Z29) (HHV-6 variant B) (Human B lymphotropic virus) | Glycoprotein U47 | TPILNAHTWEKSTTP |
| HHV6 8 | 69964 | Human herpesvirus 6B (strain Z29) (HHV-6 variant B) (Human B lymphotropic virus) | Glycoprotein U47 | TVKTFKEDRFQRTTLKSSSE |
| HHV6 9 | 101322 | Human herpesvirus 6B (strain Z29) (HHV-6 variant B) (Human B lymphotropic virus) | Large structural phosphoprotein (100 kDa phosphoprotein) (pp100) (Major antigenic structural protein) | RKQKKLDLLGSWTKEKNDKA |
| HHV7 1 | 68152 | Human herpesvirus 7 (strain JI) (HHV-7) (Human T lymphotropic virus) | Large structural phosphoprotein (100 kDa phosphoprotein) (pp100) | LHNSNKVIDIVSSPK |
| HHV7 2 | 68153 | Human herpesvirus 7 (strain JI) (HHV-7) (Human T lymphotropic virus) | Large structural phosphoprotein (100 kDa phosphoprotein) (pp100) | LPKNKIDYHSTFFLP |
| HHV7 3 | 68724 | Human herpesvirus 7 (strain JI) (HHV-7) (Human T lymphotropic virus) | Large structural phosphoprotein (100 kDa phosphoprotein) (pp100) | SFTEKMQPVRSPFFL |
| HHV7 4 | 67866 | Human herpesvirus 7 (strain JI) (HHV-7) (Human T lymphotropic virus) | Tegument protein UL11 homolog | EEELQLEKPLLNKQD |
| HHV8 1 | 115327 | Human herpesvirus 8 (HHV-8) (Kaposi's sarcoma-associated herpesvirus) | ORF73 | EQELEEQEQELEEQEQELEEQEQELEEQEQELEEQEQELE |
| Scramble 1 | - | - | Derived from HHV1 5 | DQRLDTRPREIHHPKRRIAK |
| Scramble 2 | - | - | Derived from HHV4 5 | PRYPERQEGPADAYGEFFHVGHFPP |
| Scramble 3 | - | - | Derived from HHV5 10 | PMDAWSNGTGPPALP |

**Supplementary Table S3. Commercial viral antigens used in assay development and validation**

| **Antigen** | **Virus** | **Vendor** | **Catalogue Number** |
| --- | --- | --- | --- |
| NSP16 | SARS-CoV-2 | MyBioSource | MBS156017 |
| ORF7A | SARS-CoV-2 | ProSci | 10-435 |
| ORF8 | SARS-CoV-2 | ProSci | 10-002 |
| NSP7 | SARS-CoV-2 | OriGene | TP750200 |
| NSP8 | SARS-CoV-2 | ProSci | 10-415 |
| NSP9 | SARS-CoV-2 | ProSci | 10-417 |
| NSP12 | SARS-CoV-2 | R&D Systems | 10686-CV |
| 3CLpro | SARS-CoV-2 | Sino Biological | 40594-V56E |
| NSP13 | SARS-CoV-2 | ProSci | 10-427 |
| NSP15 | SARS-CoV-2 | ProSci | 20-207 |
| NSP10 | SARS-CoV-2 | ProSci | 10-408 |
| NSP2 | SARS-CoV-2 | ProSci | 10-425 |
| NSP3 | SARS-CoV-2 | ProSci | 10-406 |
| Spike receptor-binding domain (RBD) | SARS-CoV-2 | Sino Biological | 40592-V08B |
| Spike S1 | SARS-CoV-2 | ProSci | 97-087 |
| Spike S2 ectodomain | SARS-CoV-2 | ProSci | 10-115 |
| Nucleocapsid | SARS-CoV-2 | ProSci | 10-434 |
| Envelope | SARS-CoV-2 | ProSci | 97-082 |
| Membrane | SARS-CoV-2 | ProSci | 10-429 |
| Helicase | SARS-CoV-2 | Sino Biological | 40596-V07E |
| RNA-dependent RNA polymerase | SARS-CoV-2 | Sino Biological | 40595-V08B |
| Methyltransferase | SARS-CoV-2 | Sino Biological | 40598-V07E |
| PLpro | SARS-CoV-2 | Sino Biological | 40593-V07E |
| ORF6 | SARS-CoV-2 | ProSci | 20-193 |
| RBD | SARS-CoV-1 | Sino Biological | 40150-V08B2 |
| RBD | MERS-CoV | Sino Biological | 40071-V08B1 |
| HBsAg | Hepatitis B virus | MyBioSource | MBS142509 |
| Glycoprotein E | Varicella-zoster virus | MyBioSource | MBS553197 |
| VP1 capsid protein | Enterovirus 71 | BioMART | Custom request |
| Nucleoprotein | Mumps virus | ProSpec | MMP-001 |
| E1 protein | Rubella virus | ProSpec | RUB-291 |
| Nucleoprotein | Measles virus | MyBioSource | MBS319759 |
| gp64 | Autographa californica nucleopolyhedrovirus | Sino Biological | 40496-V08B |
| VP1 | Rhinovirus | MyBioSource | MBS1220686 |
| Hemagglutinin-neuraminidase | Parainfluenza virus | Sino Biological | 40629-V07B |
| Glycoprotein G | Herpes simplex virus | MyBioSource | MBS145445 |
| Glycoprotein B | Cytomegalovirus | ProSpec | CMV-211 |
| Glycoprotein G | Human metapneumovirus | Sino Biological | 40791-V08H |

**Supplementary Table S4. Prevalence of peptide-specific IgG and IgA reactivity above the positivity threshold (3000 MFI) in LC+ and LC− participants from the RECOVER cohort.**

| **Peptide** | **LC+_IgG** | **LC-_IgG** | **LC+_IgA** | **LC-_IgA** |
| --- | --- | --- | --- | --- |
| **HHV 1 1** | 54.0 | 45.8 | 24.2 | 8.4 |
| **HHV 1 2** | 49.2 | 33.7 | 27.4 | 19.3 |
| **HHV 1 3** | 44.4 | 31.3 | 12.9 | 4.8 |
| **HHV 1 4** | 68.5 | 56.6 | 28.2 | 26.5 |
| **HHV 1 5** | 53.2 | 47.0 | 17.7 | 9.6 |
| **HHV 1 6** | 37.9 | 28.9 | 16.1 | 9.6 |
| **HHV 1 7** | 39.5 | 28.9 | 16.1 | 10.8 |
| **HHV 1 8** | 42.7 | 37.3 | 22.6 | 12.0 |
| **HHV 1 9** | 42.7 | 34.9 | 17.7 | 10.8 |
| **HHV 2 1** | 33.9 | 22.9 | 14.5 | 6.0 |
| **HHV 2 2** | 59.7 | 56.6 | 25.8 | 15.7 |
| **HHV 2 3** | 38.7 | 30.1 | 20.2 | 6.0 |
| **HHV 2 4** | 36.3 | 28.9 | 6.5 | 2.4 |
| **HHV 2 5** | 40.3 | 32.5 | 14.5 | 10.8 |
| **HHV 2 6** | 42.7 | 36.1 | 14.5 | 4.8 |
| **HHV 2 7** | 42.7 | 33.7 | 24.2 | 13.3 |
| **HHV 2 8** | 28.2 | 22.9 | 4.0 | 1.2 |
| **HHV 3 1** | 61.3 | 59.0 | 33.1 | 18.1 |
| **HHV 3 2** | 24.2 | 19.3 | 4.0 | 0.0 |
| **HHV 3 3** | 51.6 | 45.8 | 44.4 | 27.7 |
| **HHV 3 4** | 79.0 | 69.9 | 29.8 | 22.9 |
| **HHV 3 5** | 34.7 | 34.9 | 17.7 | 18.1 |
| **HHV 4 1** | 68.5 | 66.3 | 26.6 | 13.3 |
| **HHV 4 2** | 35.5 | 38.6 | 3.2 | 1.2 |
| **HHV 4 3** | 74.2 | 73.5 | 26.6 | 13.3 |
| **HHV 4 4** | 62.9 | 57.8 | 14.5 | 6.0 |
| **HHV 4 5** | 82.3 | 80.7 | 18.5 | 9.6 |
| **HHV 4 6** | 64.5 | 57.8 | 12.9 | 7.2 |
| **HHV 4 7** | 57.3 | 57.8 | 20.2 | 12.0 |
| **HHV 4 8** | 56.5 | 51.8 | 13.7 | 4.8 |
| **HHV 4 9** | 66.9 | 62.7 | 29.8 | 15.7 |
| **HHV 4 10** | 83.9 | 78.3 | 29.8 | 21.7 |
| **HHV 4 11** | 24.2 | 13.3 | 8.1 | 2.4 |
| **HHV 4 12** | 58.1 | 53.0 | 8.9 | 3.6 |
| **HHV 4 13** | 33.9 | 28.9 | 3.2 | 2.4 |
| **HHV 4 14** | 45.2 | 36.1 | 4.8 | 2.4 |
| **HHV 4 15** | 75.0 | 73.5 | 75.0 | 65.1 |
| **HHV 4 16** | 60.5 | 51.8 | 26.6 | 19.3 |
| **HHV 4 17** | 58.1 | 49.4 | 8.1 | 4.8 |
| **HHV 4 18** | 69.4 | 69.9 | 45.2 | 34.9 |
| **HHV 4 19** | 69.4 | 68.7 | 18.5 | 2.4 |
| **HHV 4 20** | 59.7 | 53.0 | 20.2 | 7.2 |
| **HHV 5 1** | 37.1 | 26.5 | 8.1 | 6.0 |
| **HHV 5 2** | 35.5 | 33.7 | 12.1 | 7.2 |
| **HHV 5 3** | 35.5 | 32.5 | 10.5 | 4.8 |
| **HHV 5 4** | 38.7 | 34.9 | 4.8 | 0.0 |
| **HHV 5 5** | 34.7 | 30.1 | 6.5 | 1.2 |
| **HHV 5 6** | 46.8 | 47.0 | 42.7 | 19.3 |
| **HHV 5 7** | 45.2 | 42.2 | 25.0 | 10.8 |
| **HHV 5 8** | 50.0 | 45.8 | 12.9 | 2.4 |
| **HHV 5 9** | 46.8 | 39.8 | 30.6 | 27.7 |
| **HHV 5 10** | 37.1 | 32.5 | 5.6 | 2.4 |
| **HHV 5 11** | 37.1 | 37.3 | 17.7 | 10.8 |
| **HHV 5 12** | 33.9 | 26.5 | 7.3 | 2.4 |
| **HHV 5 13** | 22.6 | 24.1 | 1.6 | 0.0 |
| **HHV 5 14** | 26.6 | 25.3 | 6.5 | 0.0 |
| **HHV 5 15** | 22.6 | 21.7 | 4.0 | 1.2 |
| **HHV 5 16** | 36.3 | 32.5 | 8.9 | 7.2 |
| **HHV 5 17** | 38.7 | 37.3 | 10.5 | 4.8 |
| **HHV 5 18** | 35.5 | 34.9 | 4.0 | 2.4 |
| **HHV 5 19** | 40.3 | 32.5 | 16.1 | 6.0 |
| **HHV 5 20** | 33.1 | 24.1 | 2.4 | 4.8 |
| **HHV 5 21** | 36.3 | 24.1 | 12.9 | 4.8 |
| **HHV 5 22** | 22.6 | 14.5 | 3.2 | 0.0 |
| **HHV 5 23** | 32.3 | 26.5 | 4.8 | 2.4 |
| **HHV 5 24** | 40.3 | 32.5 | 23.4 | 18.1 |
| **HHV 5 25** | 30.6 | 26.5 | 9.7 | 4.8 |
| **HHV 6 1** | 45.2 | 32.5 | 29.0 | 21.7 |
| **HHV 6 2** | 68.5 | 62.7 | 48.4 | 38.6 |
| **HHV 6 3** | 25.8 | 15.7 | 20.2 | 13.3 |
| **HHV 6 4** | 45.2 | 49.4 | 27.4 | 22.9 |
| **HHV 6 5** | 52.4 | 36.1 | 37.1 | 30.1 |
| **HHV 6 6** | 61.3 | 55.4 | 56.5 | 47.0 |
| **HHV 6 7** | 66.9 | 63.9 | 48.4 | 36.1 |
| **HHV 6 8** | 37.9 | 28.9 | 29.8 | 21.7 |
| **HHV 6 9** | 43.5 | 38.6 | 27.4 | 24.1 |
| **HHV 7 1** | 24.2 | 12.0 | 9.7 | 4.8 |
| **HHV 7 2** | 33.1 | 24.1 | 5.6 | 3.6 |
| **HHV 7 3** | 16.9 | 13.3 | 9.7 | 2.4 |
| **HHV 7 4** | 69.4 | 53.0 | 10.5 | 7.2 |
| **HHV 8 1** | 55.6 | 50.6 | 11.3 | 3.6 |
| **IRR 1 6** | 7.3 | 4.8 | 4.0 | 0.0 |
| **IRR 4 5** | 25.0 | 20.5 | 8.9 | 8.4 |
| **IRR 5 11** | 0.0 | 0.0 | 1.6 | 2.4 |
