# Supplementary material for: Early HHV-6 IgG and IgA responses during acute SARS-CoV-2 infection in relation to long COVID: a multicohort observational study
Source: eBioMedicine. 2026 Aug 27;131:106455. doi: 10.1016/j.ebiom.2026.106455 (PMC13544345; doi:10.1016/j.ebiom.2026.106455)
Supplement: Supplementary file 2 [file mmc2.docx]

**SUPPLEMENTARY FIGURES**

**Supplementary Fig. S1. Validation of Isotype-Specific Detection.** Representative dilution-series experiments demonstrating secondary-antibody reactivity profiles for IgA and IgM detection in plasma samples from patients with very early acute COVID-19 selected to exhibit isotype-specific antibody responses.

**Supplementary Fig. S2. Age associations of HHV-1 and HHV-2 IgG factor scores.** Scatter plots show factor values as a function of age for HHV-1 IgG and HHV-2 IgG modules, stratified by sex at birth, with fitted linear regression lines. Spearman correlation coefficients (*ρ*), nominal *P* values, and Benjamini-Hochberg-adjusted *q* values are shown.
