# Supplementary material for: Early HHV-6 IgG and IgA responses during acute SARS-CoV-2 infection in relation to long COVID: a multicohort observational study
Source: eBioMedicine. 2026 Aug 27;131:106455. doi: 10.1016/j.ebiom.2026.106455 (PMC13544345; doi:10.1016/j.ebiom.2026.106455)
Supplement: Supplementary file 3 [file mmc3.docx]

**SUPPLEMENTARY METHODS AND MATERIALS**

**Peptide conjugation to magnetic Luminex beads**

Antibody profiling was performed using a magnetic microbead-based multiplex assay adapted from established bead-based serologic immunoassay protocols, with modifications for peptide antigens and to reduce nonspecific binding.^35–37^ During assay development, three peptide immobilisation strategies were evaluated: direct EDC/Sulfo-NHS carbodiimide coupling, adipic acid dihydrazide (ADH)-mediated spacer-based conjugation, and antibody-mediated immobilisation via N-terminal FLAG tags. Across validation experiments, ADH-mediated conjugation produced higher and more reproducible mean fluorescence intensity (MFI) values than direct carbodiimide coupling or antibody-mediated immobilisation and was therefore selected for all subsequent experiments.

Peptides were covalently coupled to carboxylated magnetic Luminex beads (MagPlex-C, Luminex Corp.) using an ADH-mediated conjugation strategy adapted from standard EDC/Sulfo-NHS chemistry, with modifications.^36,37^ Two MES buffers were used throughout the procedure: 0.1 M MES, pH 6.0 (MES I), and 0.1 M MES, pH 4.5 (MES II). For each conjugation reaction, 240 μL of stock bead suspension was transferred per well, and storage buffer was removed by magnetic separation. Beads were washed three times with 250 μL MES I.

For ADH derivatisation, beads were resuspended in 216 μL ADH (35 mg/mL in MES I), followed by 43 μL EDC (200 mg/mL in MES I). Reactions were incubated for 2 h at room temperature with gentle agitation to generate hydrazide-functionalised bead surfaces. Beads were then washed four times with 250 μL MES II and resuspended in 150 μL MES I.

For peptide coupling, 30 μg of the designated peptide was added to the ADH-modified beads, followed by 30 μL EDC (50 mg/mL in MES I) and 30 μL Sulfo-NHS (50 mg/mL in MES I). Coupling reactions were incubated for 4.5 h at room temperature, extending beyond standard reaction times to enhance coupling efficiency for peptide antigens. Beads were then washed twice with MES I.

To reduce nonspecific binding to the bead surface, coupled beads were incubated with 150 μL MES I containing 350 μg of the coupling peptide for 1 h at room temperature and then for an additional 48 h at 4°C. Bovine serum albumin (BSA) was excluded from coupling and assay buffers, consistent with prior reports that BSA can contribute to false-positive binding in Luminex-based serologic assays.^35^ After coupling, beads were pooled, aliquoted in batches, and stored in StabilGuard (Surmodics) at −80°C protected from light until use.

Peptides were synthesised with terminal modifications to improve solubility, support bead coupling, and enable display validation. For FLAG-tagged peptides, bead-bound peptides were probed with monoclonal anti-FLAG antibody (clone M1; generously provided by Dr. Brian Kobilka’s laboratory, Stanford University), and signal was measured on a Luminex FlexMap3D instrument. Antigen specificity was further evaluated using competitive inhibition assays, in which sera were preincubated with excess soluble cognate peptide before bead incubation. Blocking efficiency was quantified as the percent reduction in MFI relative to matched unblocked samples. Peptide-only and bare-bead controls were included to define baseline fluorescence.
