# Supplementary material for: Early HHV-6 IgG and IgA responses during acute SARS-CoV-2 infection in relation to long COVID: a multicohort observational study
Source: eBioMedicine. 2026 Aug 27;131:106455. doi: 10.1016/j.ebiom.2026.106455 (PMC13544345; doi:10.1016/j.ebiom.2026.106455)
Supplement: Supplementary file 4 [file mmc4.docx]

### **REAGENT VALIDATION**

| **Reagent Type** | **Reagent** | **Vendor** | **Catalogue Number** | **RRID** |
| --- | --- | --- | --- | --- |
| Detection antibody | PE-conjugated goat anti-human IgA | SouthernBiotech | 2050-09 | AB_2795707 |
| Detection antibody | PE-conjugated goat anti-human IgM | SouthernBiotech | 2020-09 | AB_2795606 |
| Detection antibody | PE-conjugated goat anti-human IgG | Jackson ImmunoResearch | 109-116-098 | AB_2337678 |
| Beads | MagPlex Microspheres, Carboxylated Magnetic Beads | Luminex | MC100XX-01 | RRID not available |
| Coupling reagent | Adipic acid dihydrazide (ADH) | Santa Cruz Biotechnology | sc-257072 | RRID not available |
| Coupling reagent | EDC hydrochloride | Thermo Fisher Scientific | 22980 | RRID not available |
| Coupling reagent | Sulfo-NHS | Thermo Fisher Scientific | 24510 | RRID not available |
| Assay stabilizer | StabilGuard Immunoassay Stabilizer | Surmodics | SG01 | RRID not available |
| Assay platform | FLEXMAP 3D Instrument | Luminex | FLEXMAP 3D | RRID not available |
